# The Therapeutic Home Environment for Chronic Diseases: A Transdisciplinary Ecosystem for Achieving Migraine Freedom and Managing Comorbid Anxiety, Insomnia, and Chronic Pain

**DOI:** 10.3390/healthcare14091123

**Published:** 2026-04-22

**Authors:** Dorothy Day Huntsman, Desiree Jenkinson, Grzegorz Bulaj

**Affiliations:** 1 Dayhouse Studio, Salt Lake City, UT 84106, USA; 2 Jenkinson Consulting, Salt Lake City, UT 84106, USA; 3 OMNI Self-Care LLC, Salt Lake City, UT 84106, USA; 4 Department of Medicinal Chemistry, College of Pharmacy, University of Utah, Salt Lake City, UT 84112, USA

**Keywords:** multidisciplinary care, precision medicine, prophylaxis, treatment-resistant, health-related quality of life, digital therapeutics, built environment, interior design, neuroarchitecture, housing

## Abstract

Home has been recognized as a health infrastructure through hospital-at-home, home care, and direct-to-consumer wellness and fitness products. However, the patient home environment has been largely overlooked by healthcare as a means to improve therapy outcomes for difficult-to-treat chronic conditions, such as migraine; high-impact pain; and treatment-resistant depression, anxiety, or insomnia. Growing research evidence enables the formulation of a therapeutic home environment standard consisting of three pillars: biophilic design, indoor environmental quality, and intentional self-care spaces that serve as habit cues and foster sleep hygiene, stress management, relaxation, physical activity, and social interactions. Together, these environmental and behavioral interventions can transform real-world inputs into clinical benefits through autonomic, circadian, and emotional regulation. We also highlight the converging roles of self-management, self-efficacy, self-regulation, and self-compassion in sustaining patient engagement and healing at home. The applicability of the therapeutic home environment as an adjunct is illustrated in the case of chronic migraine, a debilitating neurological condition commonly associated with comorbidities. Current challenges in achieving migraine freedom with FDA-approved pharmacotherapies, neuromodulation devices, and digital health technologies are underscored by the high prevalence of refractory, chronic, episodic, and pediatric migraine. Perspectives on developing a personalized, multimodal cure for migraine are illustrated through a hypothetical drug + digital combination therapy comprising anti-CGRP drugs and an AI-powered digital health platform that promotes daily self-care practices within the therapeutic home environments. In conclusion, achieving sustained freedom from high-morbidity conditions requires end-to-end care ecosystems that integrate pharmacological, cognitive, behavioral, and environmental interventions into real-world settings.

## 1. Introduction

The chronic disease crisis encompasses diverse, multifactorial disorders, ranging from cardiometabolic conditions to debilitating neurological and mental disorders. Many of these conditions are caused or exacerbated by behavioral and environmental factors, making them partly preventable. Despite advances in pharmaceuticals, biologics, medical devices, and digital therapeutics, over 50 million people in the United States (US) live with high-impact chronic pain, treatment-resistant depression or anxiety, chronic migraine, or insomnia (Table 1). This statistic does not include millions living with schizophrenia, autoimmune, and neurodegenerative disorders. Billions of people worldwide are affected by chronic diseases, underscoring their high global prevalence [1]. The impact of these disabling conditions extends beyond individuals to families, workplaces, healthcare systems, and society at large.

The patient home environment has been largely overlooked and underutilized by healthcare to improve chronic care outcomes. Our recent work introduced opportunities to create health-centric home spaces for people living with chronic pain, migraine, anxiety, depression, cancer, and other conditions, as well as pathways to scale therapeutic home environments through digital health technologies [13,14,15]. In this perspective article, we synthesize the existing literature through a narrative review and conceptual framework to position the therapeutic home environment as an integral component of multimodal chronic care comprising pharmacological, behavioral, and environmental interventions. PubMed and Google Scholar were used for keyword-based literature searches to identify relevant studies. In the subsequent sections, we define the therapeutic home environment and its three pillars, and systematically link each pillar to mechanistic pathways and associated health outcomes relevant to chronic care. Using chronic migraine and its comorbidities as an example, we propose an evidence-based approach that links daily living inputs to long-term clinical benefits through autonomic, circadian, and emotional regulation. This work aims to catalyze innovation in home-based healthcare solutions to improve outcomes in chronic disease management.

## 2. Defining the Therapeutic Home Environment

**Figure 1:**
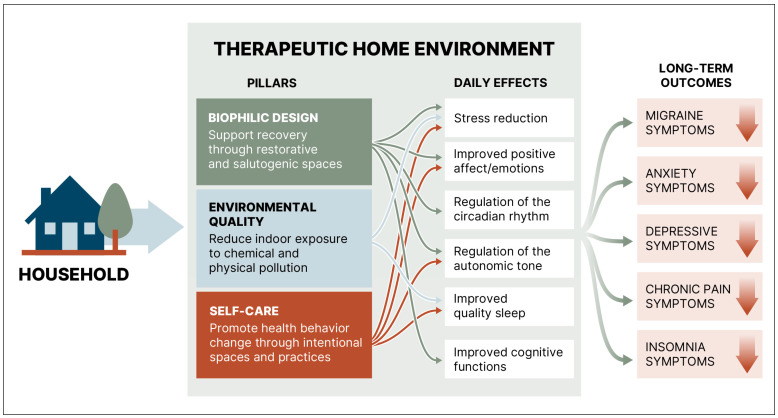
Three pillars of the therapeutic home environment for people living with chronic conditions. The proposed integration of biophilic design, indoor environmental quality, and self-care can collectively support autonomic, circadian and emotional regulation, yielding interconnected effects that can translate into long-term patient outcomes.

The therapeutic home environment comprises the physical and functional features of a home together with the behaviors and affective states they support, forming a contextual ecosystem that can improve outcomes for people with, or at risk for, chronic conditions. Building on prior work, it aims to deliver clinical benefits by integrating three pillars: (a) biophilic design, (b) the indoor environmental quality, and (c) intentional spaces coupled with self-care practices [13,14]. Biophilic design is grounded in salutogenesis and the Restoration Attention Theory and provides health benefits by enhancing exposure to nature [16,17,18,19,20]. The indoor environmental quality (IEQ) comprises artificial lighting systems and acoustics, thermal comfort and air quality [21,22,23,24]. Living spaces that facilitate self-care can help sustain long-term engagement in self-management practices and support resulting health benefits [13,25,26,27,28,29,30]. As shown in Figure 1, these three pillars can generate neurophysiological and psychological effects that collectively lead to symptom reduction through autonomic, circadian, and emotional regulation, aligning with the concept of the optimal healing environment [31]. In the subsequent sections, we formulate the therapeutic home environment by delineating health-centric features of biophilic design, the environmental quality and intentional living spaces.

Although the home is recognized as a healthcare infrastructure through hospital-at-home, home care services and wellness at home programs, the therapeutic home environment is a new approach applicable to diverse chronic conditions, including neurological and mental disorders, cardiometabolic diseases and cancer [14]. Table 2 describes key differences among diverse home-based health care ecosystems with respect to goals and approaches. It is important to emphasize that the therapeutic home environment can function both as an adjunctive modality for chronic disease management and as an integrated component of other home-based care models, including hospital-at-home, home care and wellness programs.

## 3. Biophilic Home Environment as a Modulator of Autonomic, Circadian, and Emotional Regulation

Biophilic design is an approach to architecture and interior design that integrates natural elements into the built environment to emulate the experience of being in nature [19]. It seeks to restore the human–nature connection within built environments through the direct integration of natural ecosystems, as well as sensory and spatial cues that evoke natural settings. Biophilic design principles fall into three categories: (I) nature in the space (e.g., visual, tactile, and auditory connections to nature, dynamic lighting, water features), (II) natural analogues (e.g., biomorphic and fractal patterns, natural materials such as wood and stone), and (III) nature of the space (e.g., layouts that evoke refuge, prospect, and awe) [17]. Research studies on biophilic design have been mostly focused on healthcare, workplace and educational spaces, and, to a lesser extent, on residential buildings [32]. Practical guides to biophilic design allow professionals and laypeople to apply biophilic design principles and patterns to diverse indoor environments [19,33,34,35,36].

Indoor exposure to nature elicits diverse neuronal and physiological responses that support mental and physical health and healing [17,18,37,38,39,40,41]. A recent review of 108 neuroimaging studies found that exposure to natural environments modulates large-scale brain networks (the default mode, salience, and attentional networks), shifting neural activity toward states associated with reduced stress, restored attention, and emotional regulation [41]. Biophilic design in healthcare facilities, such as hospitals and clinics, can improve patient outcomes, including reduced stress, mortality and hospitalization time [16,20,42,43,44,45,46]. From a chronic care perspective, biophilic features at home can reduce stress, enhance recovery, activate the parasympathetic nervous system, support circadian regulation, and improve mood [13,14,15]. A recent pilot study suggests that biophilic architecture may also influence neuroinflammation [47], consistent with findings that exposure to nature elicits anti-inflammatory effects [48,49,50].

Table 3 summarizes biophilic design features that together create restorative environments associated with reduced psychophysiological stress, parasympathetic activation, improved mood, and fewer depressive symptoms. These effects are directly relevant to therapy outcomes, as chronic stress is known to cause and exacerbate anxiety, depression and other morbidities. Optimizing the quality of the biophilic home environment is therefore important for maximizing health outcomes. It is noteworthy that several rating scales can be used to assess biophilic qualities in indoor spaces [34,51,52,53,54,55], while new technologies are being developed to quantify exposure to biophilic spaces [56].

**Figure 2:**
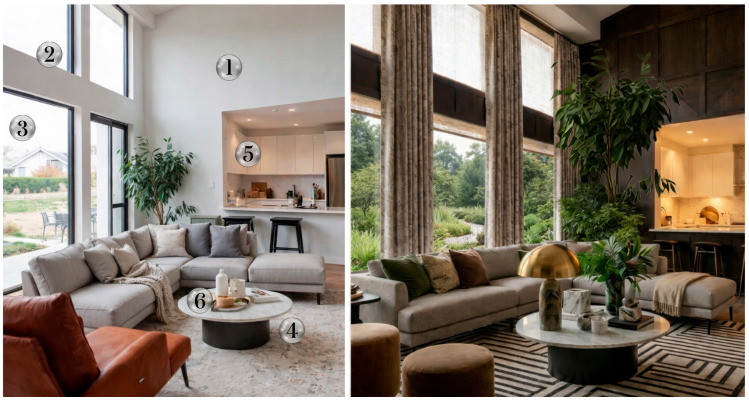
Biophilic transformation of a living room illustrating how individual design features can create a restorative environment to enhance activation of the parasympathetic nervous system, improve positive emotions and recovery from stress, and optimize exposure to natural light and outdoor nature. Design changes: (1) natural wood surface providing visual connection with nature and acoustic insulation, (2) adjustable window treatments for precision lighting control, (3) a nature view from the window, (4) fractal carpet made from natural materials providing tactile experience and acoustic insulation, (5) warm and diffuse lighting, and (6) indoor plants and flowers.

As shown in Table 3, biophilic interventions range from nature-inspired wall finishes and dynamic lighting to adding wooden furniture, biomorphic features, and indoor plants. In practice, people living with chronic conditions may prioritize the bedroom and main living room for biophilic upgrades. Figure 2 illustrates an example of transforming a living room into a restorative, biophilic space. Layering natural wood surfaces, biomorphic patterns, indoor and outdoor greenery, and warm, tactile materials is intended to reduce stress and promote a sense of safety and calm. Adjustable window treatments and smart circadian lighting regulate natural and artificial light exposure, supporting circadian rhythm while minimizing sensory overload. In addition, an essential oil diffuser can provide the benefits of aromatherapy, while a HEPA air purifier and acoustic panels mitigate air and noise pollution, respectively. Together, these design features convert the space from a neutral backdrop into a daily living space that actively supports well-being and health outcomes.

## 4. Optimizing Indoor Environmental Quality for Chronic Disease Management

The built environment can either support health or contribute to disease, a concept recognized in the literature and practice [23,117,118,119,120]. Table 4 provides examples of the indoor environmental features that can contribute to and exacerbate symptoms, or may improve therapy outcomes. The optimal therapeutic home environment for people with chronic conditions aims to reduce exposure to harmful chemical and physical factors while improving IEQ features that actively support healing (for examples of environmental pollution that negatively impact household residents, see Table 2 in [14]). Enhancing indoor air quality is known to influence COPD and asthma outcomes [121,122], but it is less appreciated that it promotes quality sleep and brain health [123]. One often overlooked opportunity to improve health outcomes through the home environment is optimized lighting [24,72,73,74,124,125]. Another overlooked aspect is daily exposure to noise pollution that can disrupt sleep and health-related quality of life [126,127]. Overall, it is essential to design and maintain indoor environments that consistently support healing.

It is important to note that the health benefits of optimized indoor environmental quality can be influenced by the outdoor environment and neighborhood conditions that affect physical and mental health [142,145,150,179,180,181]. Long-term exposure to environmental pollution can increase the risk of migraine, depression, anxiety, and cardiovascular disease [143,144,182,183]. Living near major roads increases noise and air pollution, which can worsen stress, sleep, and cardiovascular and neurodegenerative conditions [154,184,185,186,187]. On the other hand, neighborhoods can reduce the risks of chronic conditions [59,188,189,190,191,192]. Living within 300–500 m of green space may improve mental health, reduce stress, and increase physical activity [191,193,194,195,196,197]. Neighborhoods with higher accessibility to green spaces exhibited lower mental health utilization [188]. A place of residence that has a walkable accessibility to green spaces can improve cardiovascular outcomes [198,199]. Research suggests that residential green spaces are associated with lower rates of chronic pain and other noncommunicable diseases [200,201], as well as reduced migraine and severe headaches [202]. The evidence-based guidelines for health-centric neighborhoods that prioritize urban trees and green spaces are outlined in the 3-30-300 rule [59,203].

## 5. Self-Care and Intentional Living Spaces in Chronic Disease Management

From the healthcare perspective, self-care can be defined as (a) a combination of disease self-management and self-efficacy [204], or as “the ability of individuals, families and communities to promote health, prevent disease, maintain health, and cope with illness and disability with or without the support of a health worker” [205]. Self-care and lifestyle medicine overlap because both involve daily behaviors that improve health outcomes. However, lifestyle medicine focuses on six standardized pillars, while self-care includes a broader spectrum of practices that support chronic care. Table 5 summarizes growing evidence that self-care practices—such as sleep hygiene, physical activity, stress management, nutrition, social support, music, education, and exposure to nature—can reduce symptoms and improve quality of life across several chronic conditions. These findings highlight the broad therapeutic value of home-based behavioral interventions in enhancing chronic care outcomes. For people with neurological conditions, self-care practices can provide clinical benefits and reduce the risk of comorbidities. For example, self-care and other non-pharmacological interventions have been shown to reduce migraine frequency, symptoms, and disability [206,207,208].

The home plays a central role in fostering restoration, comfort, and social connection, highlighting the importance of intentionally designed spaces within the therapeutic home environment [336]. The growing interest in self-care and lifestyle interventions creates an opportunity to transform homes into environments that support health-promoting behaviors and serve as contextual cues and facilitators of daily self-care practices. The use of interior environment cues can be effective in promoting health behavior change [337,338,339]. Dedicated spaces that foster quality sleep, physical exercise, mindfulness-based relaxation and social support serve as enablers and reminders of daily practices that can reduce disease symptoms. Table 6 provides a rationale and recommendations for creating intentional spaces that integrate biophilic design and functionalities fostering self-care.

**Figure 3:**
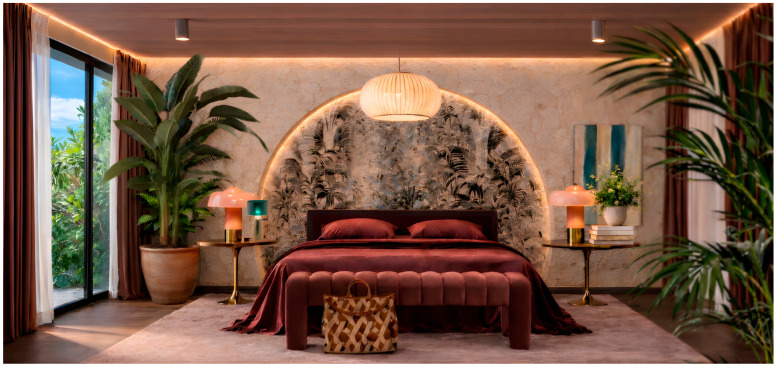
Example of an intentionally designed bedroom environment incorporating biophilic elements intended to support autonomic, circadian, and emotional regulation. Warm natural colors, indoor plants, wood and other natural materials, biomorphic patterns, and optimized morning and evening lighting create a restorative space that promotes relaxation, stress reduction, and psychological safety.

Given the importance of quality sleep for people living with chronic conditions, the bedroom should function as an intentionally optimized self-care space that integrates balanced biophilic design with precise lighting control to support circadian alignment and autonomic regulation (Figure 3). Natural materials, for instance, solid wood bed frames, breathable bedding, bedsheets, and comforters made of cotton, can enhance tactile comfort, while layered, dimmable lighting and effective window coverings minimize glare and control exposure to outdoor light. Equally important is maintaining high indoor air quality by placing a dedicated HEPA air purifier in an unobstructed area of the bedroom. Together, these features transform the bedroom into a restorative environment that serves as a cue and catalyst for fostering sleep quality.

**Figure 4:**
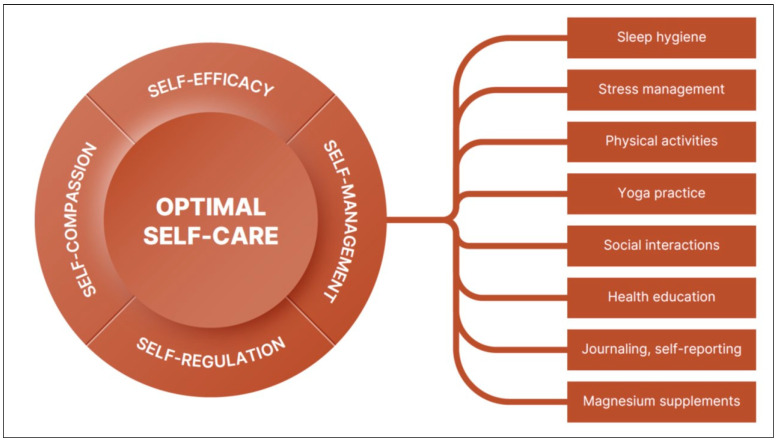
Multidimensional components of personalized self-care interventions for chronic conditions, as exemplified by managing migraine and comorbid conditions. Optimal self-care emerges from the interaction of self-management behaviors with core self-care competencies, including self-efficacy (confidence and knowledge), self-regulation (goal setting and self-monitoring), and self-compassion (self-kindness and reduced self-judgment).

Beyond intentionally designed spaces that support self-management, the long-term impact of self-care depends on core competencies: self-efficacy, self-regulation, and self-compassion. Figure 4 illustrates this multidimensional model of optimized self-care. In chronic care, self-efficacy—the belief in one’s ability to manage and improve health—can reduce headache-related disability and improve adherence to behavioral therapies [359,360,361]. Self-compassion (positive attitude toward the self) and self-regulation (the ability to manage thoughts, emotions and actions) are connected and vital for health outcomes [362]. For example, self-compassion supports health behaviors and is associated with better physical health, sleep and emotional regulation [363,364]. Self-regulation encompasses self-monitoring, feedback and goal setting to improve health behavior changes and sustained therapy outcomes [365]. When combined, the core self-care competencies and the three pillars of the therapeutic home environment form the foundation for sustained engagement in health-promoting behaviors.

## 6. Multimodal Therapeutic Ecosystems for Migraine and Associated Comorbidities

An example of a difficult-to-treat chronic condition is migraine, a debilitating disorder characterized by headaches, photophobia, phonophobia, nausea, vomiting, comorbid anxiety, depression, chronic pain and insomnia. In the US, it is estimated that over 40 million people live with migraine [2,366]. Over 2 million Americans suffer from chronic migraine, defined as having at least 15 monthly migraine days [2,367]. Approximately 5–10% of migraineurs are diagnosed with refractory migraine [4,5]. Migraine affects twice as many women as men, while the age prevalence peaks between 30 and 50 years old [368]. The prevalence of headaches, including migraine, among US children (ages 0–17) is approx. 17% [369]. Chronic migraine affects approximately 0.8% of U.S. adolescents (ages 10–19), or about 450,000 youth. [2,370]. More than 1 billion people worldwide live with migraine, which ranks as the second leading cause of disability globally [371,372].

**Figure 5:**
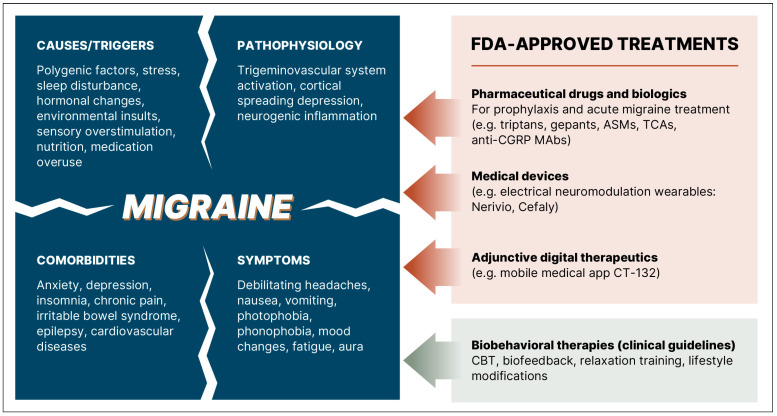
The multifactorial etiology and pathophysiology of migraine pose real-world challenges for personalized therapies that can simultaneously provide a sustained freedom from migraine and associated comorbidities. Recommended biobehavioral therapies are based on American Headache Society clinical guidelines [380]. ASMs—antiseizure medications, TCAs—tricyclic antidepressants; anti-CGRP-MAbs—monoclonal antibodies targeting Calcitonin Gene-Related Peptide; CBT—cognitive behavioral therapy.

As illustrated in Figure 5, migraine is a multifactorial neurological condition with complex etiology and pathophysiology [373]. Migraine shows strong familial aggregation, while polygenic risk scores predict both disease risk and response to pharmacotherapies. [374,375]. Lifestyle-related causes of migraine include stress, sleep disturbance, diet and physical activity [376]. Environmental pollution can also increase the risk of migraines [141]. Migraine triggers include hormonal changes, sensory overstimulation and certain foods [377]. Medication overuse is also known to exacerbate migraine headaches [378]. As a result, migraine pathophysiology includes neurogenic inflammation, trigeminal system activation and cortical spreading depression. The biopsychosocial model of migraine emphasizes the interactions between genetic susceptibility, environment, psychological and lifestyle factors that together can cause symptoms and influence therapy outcomes [379].

Migraine is understood as a network disorder of sensory processing, arising from dysfunction in neural systems regulating nociceptive and sensory inputs. The complex pathophysiology involves both peripheral and central sensitization, abnormal thalamocortical connectivity, cortical hyperexcitability, and impaired habituation, which together disrupt sensory filtering and amplify neural responses to otherwise normal stimuli [381,382,383,384]. As a result, individuals with migraine experience heightened sensitivity to environmental and internal triggers—including light, sound, stress, and sleep disruption. The pathophysiological mechanisms underlying migraine are also linked to frequent comorbidities, including anxiety, depression, chronic pain, and sleep disorders, reflecting shared neurobiological pathways such as central sensitization, dysregulated serotonergic and dopaminergic signaling, and altered pain and sensory processing networks [385,386,387]. These interactions are often bidirectional, whereby comorbid conditions can both contribute to migraine onset and arise as consequences of recurrent attacks. This complexity highlights the challenges in migraine management, which are shaped by overlapping neurological, behavioral, and environmental factors.

Migraine management is primarily focused on pharmacological treatments [388,389,390,391]. Pharmacotherapies targeting the CGRP pathway are recommended as the first-line preventive therapy for migraine [392]. However, low medication adherence rates for migraine treatment and prophylaxis illustrate limitations of drug-based therapies [393,394]. FDA-approved interventions include non-invasive neuromodulation devices, such as Cefaly and Nerivio [395,396,397]. New migraine treatments include mobile apps and digital therapeutics (DTx), as exemplified by a CT-132 mobile app (Click Therapeutics), the FDA-authorized prescription digital therapeutic (PDT) [398,399,400]. CT-132 delivers a structured 12-week behavioral program, including stress-reduction skills (e.g., breathing exercises, relaxation, mindfulness) and decision-support guidance, intended to treat migraine alongside standard acute and preventive pharmacotherapies [401]. Mobile apps for migraineurs, such as Migraine Buddy (Aptar Digital Health), enable self-management, education and tracking triggers and symptoms [402].

Table 7 shows that pharmacological, device-based, behavioral, and lifestyle interventions for migraine prevention can reduce monthly migraine days (MMD) by an average of 2–3, illustrating that the effectiveness of any individual modality might not be sufficient for those with chronic migraine experiencing over 15 MMD. This table also highlights the importance of multimodal therapies to achieve sustained migraine freedom [403,404]. While pharmacotherapies targeting the CGRP pathway are being recommended as the first-line prophylactic treatment, these medications can now be paired with prescription digital therapeutics (PDTs), such as mobile app CT-132, yielding drug + digital combination therapies [399,405,406]. Studies suggest that combining pharmacotherapy with nonpharmacological interventions can further improve migraine outcomes [353,407,408].

The main goal of migraine prevention is reaching either (a) “optimal control” (defined as less than four MMD for three consecutive months), (b) “migraine freedom” (no MMD for three consecutive months), or (c) “total freedom from headaches and associated symptoms” [403,433]. However, achieving optimal control or sustained migraine freedom in people with chronic migraine (>15 MMD) or refractory migraine remains challenging, even when prescription drugs reduce symptom severity and MMD by more than 50% [433,434,435]. The new generation of migraine prophylaxis medications is expensive and not universally covered by payers [436,437], while the current care model is insufficient with respect to the gap between migraine onset and initiation of prophylaxis treatment [438]. In addition to problems with health insurance [439,440], migraineurs also face a shortage of neurologists [441].

**Figure 6:**
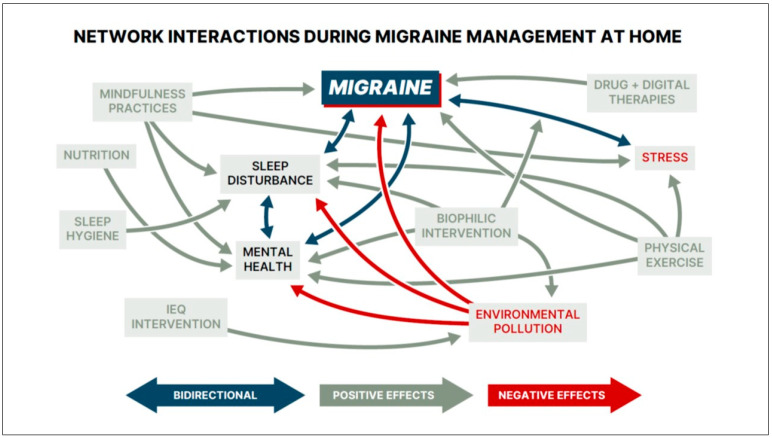
Example of real-world network interactions during migraine management at home, illustrating a broad range of daily contextual factors that can collectively influence disease outcomes. Among mental health conditions, anxiety and depression are the most prevalent comorbidities. The featured factors and their interactions are intended to capture the complexity of real-world challenges, rather than depict direct causal relationships.

Given the complex etiology of migraine and the difficulty of achieving sustained migraine freedom, the therapeutic home environment can help mitigate real-world contextual factors that influence migraine prognosis. Figure 6 shows that managing migraine and its comorbidities at home is affected by diverse, yet interconnected factors. Chronic stress, environmental pollution, and sleep disturbance can worsen symptoms, whereas combining biophilic and self-care interventions with drug + digital therapies may improve therapy outcomes. These interactions support a multimodal ecosystem in which drug + digital treatments are integrated with the therapeutic home environment.

**Figure 7:**
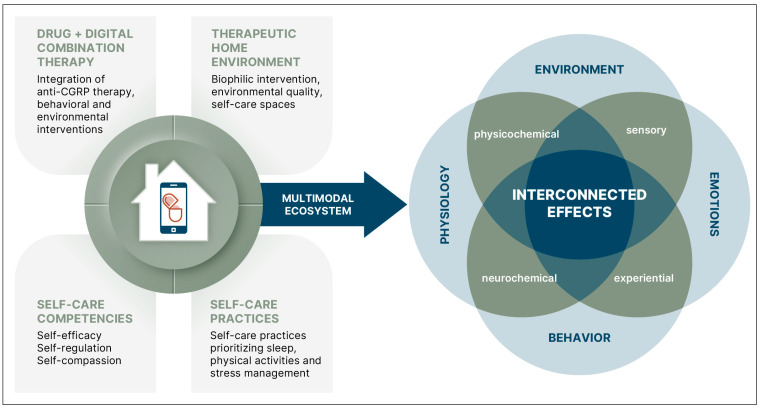
Proposed formulation of a multimodal ecosystem for migraine freedom comprising integrated pharmacological, behavioral, and environmental interventions that simultaneously modulate disease pathophysiology and contextual drivers within real-world settings. The image depicting the drug + digital + home environment combination therapy is adapted from the original Figure 2 in [15].

The multimodal cure for migraine achieves sustained migraine freedom through integrated pharmacological, behavioral, and environmental interventions that simultaneously modulate disease pathophysiology and contextual drivers. As illustrated in Figure 7, the formulation of a multimodal therapy to reach migraine freedom can include diverse active ingredients, such as anti-CGRP pharmacotherapies, cognitive behavioral therapy, health education, and personalized self-care interventions within the therapeutic home environment. For example, optimizing a biophilic bedroom and sleep hygiene, while practicing daily physical exercise and stress reduction in intentional home spaces, can support autonomic, circadian, and emotional regulation. Because autonomic, circadian, and emotional regulation converge on hypothalamic–brainstem–thalamic–limbic circuits implicated in the pathophysiology of migraine and its comorbidities, these effects provide a plausible mechanism through which biophilic interventions and self-care may reduce sensory amplification and trigger susceptibility while alleviating comorbid symptoms.

When combined with magnesium supplementation and migraine tracking, daily self-care interventions may enhance the likelihood of sustained migraine freedom. Self-care practices that can lead to reduced MMD and comorbid symptoms are shown in Table 5 and Table 7, whereas features that can be incorporated into the therapeutic home environment for migraine are listed in Table 3, Table 4 and Table 6. Integrating self-care with the three pillars of the therapeutic home environment—biophilic design, indoor environmental quality, and intentional self-care spaces—may be essential for helping individuals reach sustained freedom from migraine and comorbidities, such as chronic pain, anxiety and insomnia. We hypothesize that these therapeutic effects are mediated by downregulating sympathetic activity, enhancing parasympathetic tone, reducing central sensitization, and strengthening circadian rhythms, thereby stabilizing limbic–thalamic circuits.

We propose that integrating pharmacological, cognitive, behavioral, and therapeutic home environment interventions within a multimodal ecosystem produces interconnected physiological and experiential effects that simultaneously target shared neural circuits underlying migraine and comorbid conditions. The combination of non-pharmacological and pharmacological ingredients can be delivered via a digital health platform as drug + digital combination therapies (also known as hybrid drugs) using a regulatory strategy of adjunctive DTx, or “prescription drug use-related software” (PDURS) framework. By integrating multiple modalities within a single ecosystem, such a digital health platform addresses migraine as a network disorder in which daily physiological, behavioral, emotional, sensory, and environmental inputs collectively contribute to long-term migraine outcomes.

## 7. Technology-Enabled Implementation of Therapeutic Home Environments

The rapid expansion of AI and digital health technologies provides a pragmatic pathway for implementing and scaling therapeutic home environments. For example, therapeutic home environments can be delivered by medical-grade digital platforms that integrate e-design, e-commerce, patient education and health coaching [14]. These platforms can combine generative AI-powered interior design and visual attention software to rapidly visualize bespoke biophilic interiors, while bridging a patient’s style and affective appraisal with design principles [442,443,444]. In addition, wearable-based biofeedback can further personalize biophilic design based on emotional responses [445].

For people living with migraine, recent collaborations between Aptar Digital Health and ŌURA, and between Click Therapeutics and Ultrahuman, provide insights into an emerging chronic care model in which combining mobile apps with wearable-based biometrics and symptom tracking can improve therapy outcomes. Such mobile app + wearable combinations integrate real-time data related to sleep, stress and physical activities with actionable guidance, positioning the patient’s home as a continuous site of monitoring, engagement and behavior change. When further combined with pharmacotherapies and an optimized therapeutic home environment, biofeedback-based guidance illustrates a personalized approach to migraine management. This approach is compatible with AI-powered health coaching that supports education and disease self-management at home [446]. It is noteworthy that AI-powered digital health platforms delivering therapeutic home and behavioral interventions are also compatible with the PDURS framework, potentially yielding a multimodal, end-to-end therapeutic ecosystem for chronic conditions.

People using at-home medical devices for chronic conditions—such as Nerivio REN (Theranica, Bridgewater, MA, USA)**,** Cefaly (Cefaly Technology, Seraing, Belgium), and gammaCore (electroCore, Rockaway, NJ, USA) for migraine; Flow FL-100 tDCS (Flow Neuroscience, Malmo, Sweden) and Proliv™Rx (Neurolief, Coral Springs, FL, USA) for major depressive disorder; ActiPatch (BioElectronics, Frederick, MD, USA), and Enso (Hinge Health, San Francisco, CA, USA) for chronic musculoskeletal pain; and Modius Stress (Neurovalens, Belfast, UK) and Alpha-Stim CES (Electromedical Product International, Mineral Wells, TX, USA) for anxiety and insomnia—often rely on repeated, self-administered sessions in their living spaces. A therapeutic home environment can enhance the effectiveness of these devices by optimizing a restorative environment that supports autonomic, circadian and emotional regulation before and after each treatment.

## 8. Integration of Therapeutic Home Environments into Evolving Healthcare Models

The patient home environment remains overlooked and underused in efforts to improve chronic care outcomes. While the preceding sections focused on the mechanism and applications of the therapeutic home environment, this approach also intersects with broader shifts in healthcare delivery. Increasing emphasis on home-based continuity of care and self-management highlights the relevance of daily living environments as modifiable determinants of therapy outcomes. The therapeutic home environment does not represent a new category of care, but rather an adjunctive layer that can complement pharmacological, digital, and behavioral interventions. Situating this approach within healthcare models can help clarify its relevance to health systems and payers.

*Contextualizing the Therapeutic Home Environment Within Evolving Care Models*. The therapeutic home environment can be understood as an extension of healthcare delivery models that increasingly emphasize decentralization, continuity of care, and non-pharmacological treatments. As healthcare systems and payers respond to the growing burden of chronic disease, the home has emerged as a point of care through hospital-at-home, remote patient monitoring, and home-based specialty services. Within this landscape, the therapeutic home environment addresses an important but underexamined dimension: the role of daily environmental exposures and home-based self-care in providing clinically meaningful benefits for people living with chronic conditions. Positioning the patient home as an adjunctive intervention that complements other therapies aligns with a multidisciplinary approach to chronic disease management.

*Contributions to Healthcare Performance and Outcomes*. Chronic conditions drive high healthcare utilization, especially when symptoms and comorbidities are poorly controlled. By reducing sleep disruption, chronic stress, and environmental triggers, the therapeutic home environment may help prevent avoidable care escalation and improve long-term outcomes for people with difficult-to-treat disorders (Table 1). This approach aligns with efforts to enhance the real-world effectiveness of existing treatments. As healthcare systems increasingly operate under risk-based and bundled payment models, contextual drivers of outcomes become more consequential. By addressing environmental and behavioral factors that influence disease prognosis, the therapeutic home environment supports tertiary prevention. By enhancing autonomic, circadian, and emotional regulation, it is also compatible with the primary prevention of lifestyle-related chronic conditions.

*Patient Engagement*. Engagement and adherence are major challenges in continuity of care and chronic disease management. Behavioral science and environmental psychology indicate that physical environments can function as persistent cues that reinforce habits, self-regulation, and self-efficacy. By bridging daily living spaces with multimodal therapy, this personalized approach (Figure 7) offers a means to support long-term engagement that complements episodic clinical encounters. This is particularly relevant in consumer-driven healthcare contexts where patient experience and perceived value influence participation. Growing willingness to engage with home-based wellness and digital health technologies underscores the relevance of structured, evidence-based solutions that can bridge consumer behavior and continuity of care. These trends are further reinforced by people seeking health-related guidance from AI-powered chatbots.

By positioning the therapeutic home environment within healthcare-relevant frames, we hope its value can be recognized by all stakeholders. This contextualization also supports the broader argument that optimizing the daily living environment is a meaningful extension of multimodal chronic disease management.

## 9. Cross-Sector Value Proposition of Therapeutic Home Environments

**Figure 8:**
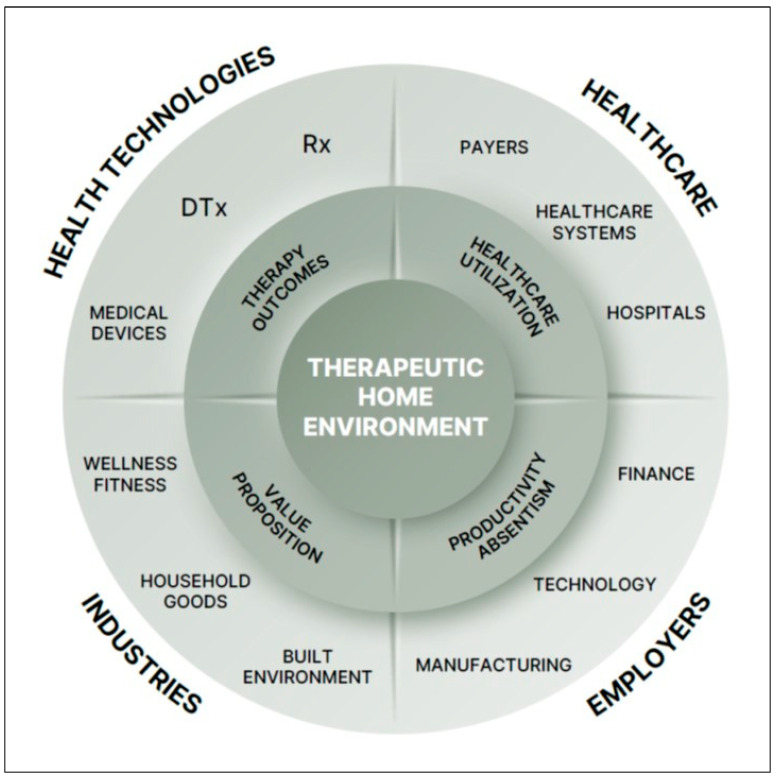
Cross-sector ecosystem illustrating the therapeutic home environment as a shared value asset connecting healthcare, consumer industries, and economic growth. Such real-world ecosystem enables bridging health outcomes, reduced healthcare utilization, productivity gains, and differentiated value propositions across diverse stakeholders.

Figure 8 illustrates the cross-sector value proposition of integrating therapeutic home environments into a real-world ecosystem that spans healthcare and consumer markets. Examples of stakeholder groups include health technology companies (e.g., pharma/biotech, medical devices, digital health), healthcare systems (payers, hospitals), employers, and household-related businesses. The win–win opportunities extend across pharma/biotech, medical devices, and digital health by enabling greater real-world effectiveness and improved patient retention. For companies such as Pfizer, AbbVie and Eli Lilly that market anti-CGRP drugs (Nurtec ODT™ (Pfizer, New York, NY, USA), Ubrelvy™ (AbbVie, North Chicago, IL, USA) and Emgality™ (Eli Lilly, Indianapolis, IN, USA), respectively), integrating these drugs with a PDURS-based digital health platform offers a compelling end-to-end scalable ecosystem to improve migraine prophylaxis.

Broad adoption and implementation of therapeutic home environments can help employers increase productivity and reduce absenteeism among employees. While this article’s focus is on the home environment, it is noteworthy that biophilic workplaces can improve stress reduction, cognitive performance and job satisfaction, while also providing restorative effects, even including improved glucose control among people with diabetes [125,447,448,449]. Finally, consumer-focused industries, e.g., real estate, home goods, wellness, and fitness, gain opportunities to differentiate products and services through health-oriented marketing and applications. By aligning financial incentives around a shared therapeutic ecosystem, integrating the therapeutic home environment can drive economic value across diverse consumer markets.

## 10. Challenges, Limitations and Future Directions

Several factors may contribute to the underutilization of a patient’s home environment as a means to improve therapy outcomes. For example, the amount and quality of research-based evidence on the therapeutic effects of biophilic design for chronic conditions remain significantly lower compared to evidence for specific self-care practices. We acknowledge that the heterogeneity of existing evidence across research areas used to formulate the therapeutic home environment is also a limitation of this work. From a healthcare perspective, integrating therapeutic home environments into clinical practice will require established guidelines based on high-quality evidence, preferably derived from systematic reviews and meta-analyses.

Closing the knowledge and knowledge-practice gaps requires more research on the long-term effects of optimized living spaces for people with specific chronic diseases. We acknowledge the difficulty of establishing causal relationships between therapeutic home environments and clinical outcomes, given the interdependent nature of environmental, behavioral, and psychosocial variables. This challenge is further compounded by the lack of validated metrics to quantify the therapeutic quality of home environments. Generating real-world evidence on the therapeutic home environment’s impact on long-term therapy outcomes requires pragmatic studies that also use decentralized RCTs, combining patient-reported outcomes with wearable-based data. Until evidence demonstrates clinically meaningful benefits—such as reducing MMD or alleviating anxiety and depressive symptoms—health insurance companies are unlikely to reimburse expenses associated with optimizing the therapeutic home environment. Adoption of the therapeutic home environment approach across countries may vary depending on national healthcare systems, clinical guidelines, and cultural and regulatory practices.

Implementation of the therapeutic home environments is determined by socioeconomic factors, such as a person’s ability to afford improved housing conditions. In addition to a person’s financial resources, optimizing the therapeutic home environment also depends on (a) personality and personal style preferences; (b) type of housing (single-family house, townhouse or multistory apartment unit); and (c) ownership, since renting limits possible home improvements. All these variables make it difficult to create a standardized formula for the therapeutic home environment that can be universally applied across all households and chronic conditions. In addition, integration of therapeutic home environments with AI-driven platforms and wearable devices raises concerns regarding data privacy, security, ownership, and regulatory compliance, which may limit adoption and scalability.

A competition with the commercial determinants of health (CDoH) presents another challenge for therapeutic home environments to provide clinical benefits, in particular for people who struggle with difficult-to-treat neurological and mental disorders. CDoH can negatively impact self-regulation, behavior change, circadian rhythms, and therapy outcomes through persuasive marketing and exposure to ultra-processed foods and alcohol, digital overstimulation, chronic stress, and sedentary lifestyle. This is particularly relevant for long-term outcomes, as sustained adherence to behavioral and environmental interventions remains a real-world challenge, with engagement in disease self-management and behavior change often declining over time.

Future studies focusing on the underlying mechanisms of biophilic design, particularly in combination with self-care practices, could deepen understanding of their therapeutic effects. While we highlight opportunities to improve outcomes for chronic migraine, future research should also evaluate the generalizability of therapeutic home environment interventions across diverse migraine populations, including differences in age, sex, socioeconomic status, comorbidity profiles, and housing contexts. Of equal importance are studies investigating the effectiveness of therapeutic home environment interventions for individuals living with chronic pain, anxiety, depression, and other chronic conditions.

Future directions should also leverage digital health platforms that unify behavior change, engagement, continuous monitoring, therapeutic home environments, and longitudinal data integration into a single ecosystem to address the gap between treatment efficacy and real-world effectiveness. Such an approach could be driven by either pharmaceutical companies through PDURS-enhanced pharmacotherapies or value-based healthcare systems focused on improving patient outcomes through tertiary prevention.

## 11. Conclusions

Over 50 million people in the US struggle with difficult-to-treat conditions, such as chronic migraine, high-impact chronic pain, and treatment-resistant depression or anxiety, highlighting real-world limitations of pharmacy care alone. Globally, chronic diseases affect a substantially larger population. Transforming households into therapeutic spaces remains an overlooked frontier in multidisciplinary chronic disease management and lags behind substantial advances in pharmacological, digital health, and medical device-based interventions.

This perspective exemplified migraine and its comorbidities to define the therapeutic home environment as an important missing link in the end-to-end chronic care ecosystem. By integrating biophilic design, optimized indoor environmental quality, and intentional spaces fostering self-care, therapeutic home environments can enhance real-world effectiveness of existing therapies for neurological, mental, neurodevelopmental, autoimmune and cardiometabolic disorders, as well as cancer, chronic infections, and others.

Although this article focuses on difficult-to-treat chronic conditions and primarily references adult populations, the therapeutic home environment is highly relevant for children living with chronic conditions. Notably, exposure to health-supportive home environments during early childhood may also represent an opportunity for improving primary prevention of multifactorial chronic diseases. We hope this work catalyzes transdisciplinary research and encourages healthcare stakeholders to recognize the home as an integral component of holistic, patient-centered care. From a practical perspective, it also highlights opportunities for clinicians to discuss the home environment and self-care with patients as modifiable factors that may improve the management of migraine and comorbid symptoms.

## Figures and Tables

**Table 1:** Examples of debilitating and difficult-to-treat chronic conditions affecting US adults. The estimate of US adults is based on 2024 population data. The number of people living with these conditions worldwide is substantially higher. GAD—generalized anxiety disorder, MDD—major depressive disorder.

Chronic Condition	Clinical Features	Impact on Daily Functioning	Estimated Affected Adults and Prevalence
Chronic migraine	Headache occurring on ≥15 days per month for >3 months, with ≥8 days meeting migraine criteria	Unpredictability coupled with chronic stress, emotional burden, heightened environmental sensitivity	≥2.4 million (0.91% general population, [2])
Refractory migraine	Chronic migraine with persistent disability despite multiple adequate preventive treatment trials	Severe disability with significant impairment in work, social, and daily activities	1.6–2 million (5–10% of migraineurs [2,3,4,5])
High-impact chronic pain	Pain occurring on most days for ≥3 months that substantially limits daily activities	Constant limitations in daily functioning accompanied by fatigue and emotional and social burden	17–20 million, (7–8% general population [6])
Treatment-resistant generalized anxiety disorder	GAD symptoms that fail to respond to at least first-line pharmacotherapy and psychotherapy	Excessive and frequent worry accompanied by restlessness, fatigue, impaired concentration, and irritability	2.3–2.9 million (2.9–3.7% GAD prevalence 30% of GAD patients are TR [7,8,9])
Treatment-resistant depression	MDD symptoms that do not respond to at least two different antidepressant classes of drugs	Persistent depressed mood, anhedonia, fatigue, cognitive and functional impairment, hopelessness, and suicidality	≥2.8 million adults (20–30% of MDD patients [10])
Chronic insomnia	Difficulty sleeping and daytime impairment occurring at least 3 nights per week for ≥3 months	Lasting exhaustion, impaired concentration, emotional dysregulation, and hyperarousal with preoccupation about sleep	26.7 million (10% general population [11,12])

**Table 2:** Differentiating home-based health care ecosystems. PA—physician assistant, AD—Alzheimer’s disease, COPD—Chronic Obstructive Pulmonary Disease.

	Therapeutic Home Environment	Hospital-at-Home	Home Care	Wellness at Home
Description	Home optimized for improved chronic care outcomes	Acute hospital-level care at home with clinical oversight and monitoring	Clinical, caregiving and personal care at home	Consumer wellness and lifestyle support at home
Goals	Reduce disease symptoms; improve effectiveness of existing therapies and self-management	Reduce hospital stays, costs and complications	Help individuals maintain their physical and functional abilities, live independently at home	Enhance well-being, healthy habits and lifestyle. Extend healthspan and longevity
Approach	Home spaces supporting autonomic, circadian, emotional, regulation and behavior change	Multidisciplinary clinical care (physicians, nurses, PAs) with in-person and telemonitoring	Professional care and therapies coordinated and delivered by an interdisciplinary team	Home-based gyms, wellness corners supporting stress management, fitness. Wellness coaching
Delivery capacity	Higher scalability due to compatibility with integrated digital health and ecommerce and consumer-led adoption	Lower scalability due to dependence on in-person clinical teams and payer-based reimbursement	Lower scalability due to dependence on logistics for in-person visits	Higher scalability due to direct-to-consumer distribution channels and compatibility with wearables and health apps
Examples of medical indications	Migraine, chronic pain, epilepsy, depression, anxiety, insomnia, cancer, dementia, Alzheimer’s and Parkinson’s disease	Pneumonia, COPD, asthma, congestive heart failure, soft-tissue and urinary tract infections, cancer (pilot programs)	Stroke rehabilitation, dementia and AD support, diabetes complications, wound care, post-surgical recovery	Primary prevention

**Table 3:** A summary of biophilic design features and recommendations for creating the therapeutic home environment for people living with chronic conditions. PNS—parasympathetic nervous system, HRQoL—health-related quality of life.

Biophilic Features	Neurophysiological Effects	Recommendations for Optimal Experience	References
Direct proximity to trees	Stress reduction, improved mood and mental health, cognition restoration	At least three mature trees visible from home, preferably from the front (entrance) and through at least one window	[57,58,59]
Nature views from the windows	Stress reduction, activation of the PNS, improved physical and mental health and psychological well-being, reduced risk of anxiety and depression	Unobstructed view of both vegetation (natural or planted landscapes) and the sky from at least one window located in the main living space; for a multistory apartment, a balcony with a planter can provide a view of outdoor greenery	[60,61,62,63,64,65,66]
Natural colors	Evoke positive, low-arousal emotions; promote relaxation	Personalized wall finish with natural calming hues, such as earthy neutrals, blues and greens	[67,68]
Dynamic and diffuse lighting	Regulation of the circadian rhythm; reduced fatigue, anxiety and depressive symptoms	Smart lighting systems mimicking daylight cycle and control illuminance; window coverings in every room to minimizes glare from sunlight	[64,69,70,71,72]
Warm light	Improve positive mood, stress reduction	Sources of warm light from the windows, floor and table lamps	[73,74]
Natural soundscapes	Stress reduction and recovery; activation of the PNS, improved mood	Indoor water feature; dedicated audio system facilitating daily exposure to nature sounds	[75,76,77,78]
Refuge/prospect space	Stress reduction, perceived safety and comfort	A comfortable lounge chaise made of natural materials and sheltered by indoor plants and side table/shelf providing a window view toward outdoor nature	[17]
Indoor plants and flowers	Stress reduction, activation of the PNS, improved mood and positive emotions	Each room has a layered arrangement of odd number of indoor plants creating focal points of 30–40% of visual weight, large (>5 ft) and medium (2–4 ft) plants potted in planters that display biomorphic patterns; living plant wall	[50,79,80,81,82,83,84]
Wood-based interiors and furniture	Stress reduction, activation of the PNS, calming prefrontal cortex, increase positive affect	Flooring and a majority of furniture (bed frames, dressers, tables, chairs, cabinets) that is made of solid wood, wood accent wall panels also serving as acoustic panels or creating a refuge space	[85,86,87,88,89,90,91]
Natural materials (other than wood)	Improve positive emotions, comfort and relaxation through multisensory (visual and haptic) connection with nature	Seating pieces upholstered in natural fabric, rugs made from wool/cotton providing relaxing underfoot texture, visual connection with nature through biomorphic patterns and improving acoustics, stone countertops creating awe	[92,93,94,95]
Biomorphic/fractal patterns and biophilic art	Reduced stress and anxiety, improved recovery from stress and relaxation	Noticeable visual weigh of biomorphic patterns through wall finishing, furniture, décor and accessories	[96,97,98,99,100,101,102,103]
Awe	Stress reduction, activation of the PNS, fostering pro-social behavior and positive emotions	Extraordinary indoor biophilic features combined with a spectacular window view of natural landscape	[17,104,105,106]
Indoor–outdoor connection with nature	Improved mental health and HRQoL	The presence of a garden within mature landscape enabling horticultural therapy; a patio/balcony offering year-round connection with nature through vegetation and biomorphic planters	[58,101,107,108,109,110,111]
Periodic scentscapes	Aromatherapy can reduce stress, anxiety, and pain, improve sleep and relieve migraine	Availability of nebulizing diffusers and high-quality essential oils (e.g., lavender) that are located in a bedroom or a living room	[112,113,114,115,116]

**Table 4:** Examples of indoor environmental quality features that influence chronic care outcomes. IAQ—indoor air quality, HVAC—heating, ventilation and air conditioning, HEPA—high efficiency particulate air, MERV—minimum efficiency reporting value.

IEQ Features	Relevance to Chronic Conditions	Recommendations for Optimal Experience	References
Exposure to natural light	Regulate circadian rhythm; improve mood and sleep; reduce fatigue, potential trigger for migraine; hypersensitivity to light	Controlling light intensity from a complete blackout to a comfortable lighting intensity through window covering and smart lighting system, including dimmers in every room; availability of migraine-centric spectacles	[24,71,128,129,130]
Exposure to warm light	Improve positive mood; stress reduction	Sources of warm light from the windows, floor and table lamps	[73,74]
Exposure to green light	Reduce migraine headaches and fibromyalgia symptoms	Presence of at least two lamps (bedroom and living room) facilitating daily exposure to green light with narrow band wavelength	[131,132,133,134]
Evening exposure to blue-spectrum light	Disruption of circadian rhythm and quality sleep due to both artificial lighting and screen exposure	Smart lighting system enabling a regulation of blue light exposure, maximizing in the morning and minimizing in the evening	[135,136]
Exposure to screen time	Disruption of circadian rhythm; impacts self-esteem and social connections; increased depressive symptoms and sedentary behaviors	Presence of Frame Smart TV that displays biophilic patterns, a dedicated smartphone charging station made of natural materials, a discreet dedicated space for computers	[137,138,139,140]
Indoor air quality	Air pollution can contribute to systemic inflammation, sleep disturbance, migraine triggers (smells)	IAQ monitoring system, CO and smoke detectors, HVAC with air filters rated MERV 13 or higher for controlling air quality, HEPA air purifiers in bedroom and a main living area	[141,142,143,144,145,146,147,148]
Natural air flow and thermal comfort	Reducing indoor air pollution, which can exceed outdoor levels; improving thermal comfort and quality sleep through ventilation	Presence of operable windows located away from traffic and industrial pollution, HVAC system; presence of circulating fans in areas difficult for ventilation	[149,150,151,152,153]
Acoustic comfort	Reduced noise pollution to prevent sleep disturbance, negative emotions; increased risks for cardiovascular and mental disorders; mitigating hypersensitivity to sound during migraine episodes	Installed acoustic panels to maintain acoustic levels in bedroom and living room on average below 40 dB, rugs and accent wood wall panels can additionally absorb excessive sound	[154,155,156]
Periodic soundscapes	Reduced stress and depressive symptoms; improved positive emotions and analgesia; reduced pro-inflammatory cytokines	Dedicated and programable audio system facilitating daily exposure to nature sounds and relaxing music	[76,157,158,159]
Ergonomics	Prevent and reduce body tension and musculoskeletal symptoms	Ergonomic seating pieces that reduce body tension	[160,161,162,163,164]
Clutter	Increased stress, decreased sleep quality	Available integrated storage solutions, furniture layout balances flow and function, tabletops avoid over-accessorizing	[165,166,167]
Dampness	Increased risk of headaches and sleep disturbance	Tested negative for dampness using moisture meter across all indoor spaces	[168,169,170]
Drinking water quality	Supports hydration while eliminating heavy metal and microplastic pollutants that cause neurotoxicity	Available point-of-use water filtration system	[171,172]
Exposure to nano- and microplastic pollution	Dysregulated immunity of the digestive system, impact on the gut microbiome, accumulation in the brain, neuroinflammation	Availability of glass containers for food storage, a majority of clothing made of natural and not synthetic textiles, personal care products in glass containers	[173,174,175,176,177,178]

**Table 5:** Examples of health outcomes associated with self-care practices related to chronic care. SR, symptom reduction; RR, reduced risk; IB, indirect benefits; QoL, quality of life.

Chronic Condition	Sleep Quality/Hygiene	Physical Exercise	Stress Management	Nutrition/Supplements	Social Support	Listening to Music	Health Education	Exposure to Nature
Migraine	SR, QoL [209,210,211]	SR [212,213,214]	SR, QoL [215,216,217]	SR [218,219,220,221]	QoL [222,223,224]	SR, QoL [225,226]	SR, QoL [217,227,228,229]	IB [145,230]
Chronic Pain	SR, QoL [231,232,233,234]	SR, QoL [235,236,237]	SR, QoL [238,239,240]	SR [241,242,243,244]	QoL [245,246,247,248,249,250]	SR, QoL [159,251]	SR, QoL [252,253,254,255]	RR, QoL [200,256,257,258]
Depression	RR [259,260,261]	SR, RR, QoL [262,263,264,265]	SR [266,267,268]	SR, RR [269,270,271,272,273]	RR, QoL [274,275,276,277]	SR [278,279,280,281]	SR [282,283,284]	SR, RR, QoL [180,285,286,287]
Anxiety	RR [288,289,290]	SR, RR [291,292,293]	SR [294,295,296,297]	SR [298,299,300]	SR, QoL [301,302,303]	SR [304,305,306]	SR, RR [307,308]	RR [180,309,310]
Insomnia/Sleep disturbance	SR [311,312]	SR, QoL [313,314,315,316]	SR [317,318,319]	SR, RR [320,321,322,323]	RR, QoL [324,325,326]	SR, QoL [327,328,329,330]	SR [331,332]	QoL [65,333,334,335]

**Table 6:** Characteristics of intentional spaces serving as contextual cues and fostering self-care within the therapeutic home environment.

Intentional Space	A Rationale Related to Chronic Care Outcomes	Recommendations for Optimal Experience	Daily Contextual Cues	References
Bedroom	Optimize sleep comfort and sleep hygiene; improve mood; reduce allostatic load and sleep disturbance	Biophilic features including natural wood bed frame; pillows, comforter, bedding sheets and medium-firm mattress made of natural materials; control of lighting (dynamic and zero-blue and green light); HEPA air purifier, acoustic panels with biomorphic patterns, aromatherapy diffuser; no electronic devices (TV/computer screens)	Associations with quiet and comfortable space dedicated to restful sleep	[340,341,342,343,344,345,346,347]
Relaxation space	Based on the biophilic principles, refuge and prospect, a place for self-regulation and mindfulness-based stress reduction	Natural rugs for defining the area; lounge chaise surrounded by indoor plants, ambient green light lamp, book and plant shelf, nearby biophilic art, accessible soundscape or noise-canceling headphones, control of natural light	Associations with safe and restorative sanctuary dedicated to rest	[17,102,348,349,350]
Wellness/Fitness space	Facilitating access to physical exercises, strength training, yoga, stretching and other activities	Presence of a dedicated wellness area that comprises treadmill or other fitness equipment that enables medium-intensity and strength exercises independent of outdoor conditions, place for yoga mat for stretching and yoga exercises	Associations with convenience of physical exercises	[351,352,353,354,355]
Space for social connections	Fostering prosocial behaviors and social support from family and friends	A dedicated area comprising 2–3 ergonomic armchairs centered around a solid wood coffee table featuring a decorative indoor plant, presence of a natural rug with biomorphic motifs that defines the social connection zone	Associations with engaging and uplifting conversations	[202,356,357,358]

**Table 7:** Examples of migraine prophylaxis modalities and their abilities to reduce monthly migraine days (MMD) in episodic and chronic migraine. MMD data are based on meta-analysis or randomized controlled trials (RCTs), and are not placebo-corrected. ASM—anti-seizure medication, CGRP—calcitonin gene-related peptide, VGCC—voltage-gated calcium channel, GABA—gamma-aminobutyric acid, NMDA—N-methyl-D-aspartate.

Intervention	MMD Reduction	Mechanism of Action	References
Rimegepant	3.6	CGRP receptor antagonist	[409]
Frenamezumab	2.2	Anti-CGRP monoclonal antibody	[410]
Botox	2	Modulation of neurotransmitter release	[411]
Topiramate	1	ASM modulation of VGCC and GABA-meditated inhibition	[412]
Propranolol	1.5	Non-selective beta-blocker	[413]
Nerivio	4	Remote electrical neuromodulation of the conditioned pain pathway	[414]
DTx CT-132 (+Rx)	3	Cognitive/behavioral intervention targeting patient responses to environmental and internal stimuli	[399]
Magnesium supplementation	1.7	Reduced hyperexcitability through modulating NMDA receptors and glutamate release, reducing neuroinflammation	[219,415,416,417,418]
Physical exercise	3.6	Modulation of pain modulatory network, hypoalgesia and autonomic tone; reduction in chronic inflammation, adaptive neuroplasticity	[351,419]
Yoga and breathing exercises	1.4	Stress reduction, modulation of autonomic and vascular tones, improving sleep quality	[420,421,422,423]
Mindfulness-based stress reduction	1.6	Stress reduction; self-regulation	[217]
Relaxation training	1	Stress reduction; self-regulation	[216]
Sleep hygiene	6.8	Regulation of circadian rhythm and hypothalamic functions	[424,425]
Education	2	Trigger awareness; improved self-efficacy and self-management	[217,426]
Migraine diary	2.4	Trigger awareness; improved locus of control and engagement	[427]
Placebo effect	1.8–5.6	Expectation, conditioning	[428,429,430,431,432]

## Data Availability

No new data were created or analyzed in this study. Data sharing is not applicable to this article.

## Abbreviations

The following abbreviations are used in this manuscript:

AI	Artificial intelligence.
CDoH	Commercial determinants of health.
CGRP	Calcitonin gene-related peptide.
DTx	Digital therapeutics.
FDA	Food and Drug Administration.
GAD	Generalized anxiety disorder.
HEPA	High-efficiency.
HRQoL	Health-related quality of life.
IEQ	Indoor environmental quality.
MMD	Monthly migraine days.
MDD	Major depressive disorder.
PNS	Parasympathetic nervous system.
PDT	Prescription digital therapeutics.
PDURS	Prescription drug use-related software.
RCT	Randomized controlled trial.
US	United States.

## References

[1] (2020). Global burden of 369 diseases and injuries in 204 countries and territories, 1990-2019: A systematic analysis for the Global Burden of Disease Study 2019. Lancet.

[2] Cohen F., Brooks C.V., Sun D., Buse D.C., Reed M.L., Fanning K.M., Lipton R.B. (2024). Prevalence and burden of migraine in the United States: A systematic review. Headache J. Head Face Pain.

[3] Ornello R., Andreou A.P., De Matteis E., Jürgens T.P., Minen M.T., Sacco S. (2024). Resistant and refractory migraine: Clinical presentation, pathophysiology, and management. eBioMedicine.

[4] Rosignoli C., Ornello R., Caponnetto V., Onofri A., Avaltroni S., Braschinsky M., Šved O., Gil-Gouveia R., Lampl C., Paungarttner J. (2024). Resistant and refractory migraine—Two different entities with different comorbidities? Results from the REFINE study. J. Headache Pain.

[5] Martelletti P., Katsarava Z., Lampl C., Magis D., Bendtsen L., Negro A., Russell M.B., Mitsikostas D.D., Jensen R.H. (2014). Refractory chronic migraine: A consensus statement on clinical definition from the European Headache Federation. J. Headache Pain.

[6] Zelaya C.E., Dahlhamer J.M., Lucas J.W., Connor E.M. (2020). Chronic pain and high-impact chronic pain among US adults, 2019. NCHS Data Brief.

[7] Ansara E.D. (2020). Management of treatment-resistant generalized anxiety disorder. Ment. Health Clin..

[8] Kessler R.C., Petukhova M., Sampson N.A., Zaslavsky A.M., Wittchen H.U. (2012). Twelve-month and lifetime prevalence and lifetime morbid risk of anxiety and mood disorders in the United States. Int. J. Methods Psychiatr. Res..

[9] Bokma W.A., Batelaan N.M., Penninx B.W.J.H., van Balkom A.J.L.M. (2021). Evaluating a dimensional approach to treatment resistance in anxiety disorders: A two-year follow-up study. J. Affect. Disord. Rep..

[10] Zhdanava M., Pilon D., Ghelerter I., Chow W., Joshi K., Lefebvre P., Sheehan J.J. (2021). The prevalence and national burden of treatment-resistant depression and major depressive disorder in the United States. J. Clin. Psychiatry.

[11] Ford E.S., Cunningham T.J., Giles W.H., Croft J.B. (2015). Trends in insomnia and excessive daytime sleepiness among U.S. adults from 2002 to 2012. Sleep Med..

[12] Morin C.M., Jarrin D.C. (2022). Epidemiology of Insomnia: Prevalence, Course, Risk Factors, and Public Health Burden. Sleep. Med. Clin..

[13] Huntsman D.D., Bulaj G. (2022). Healthy Dwelling: Design of Biophilic Interior Environments Fostering Self-Care Practices for People Living with Migraines, Chronic Pain, and Depression. Int. J. Environ. Res. Public Health.

[14] Huntsman D.D., Bulaj G. (2025). Home Environment as a Therapeutic Target for Prevention and Treatment of Chronic Diseases: Delivering Restorative Living Spaces, Patient Education and Self-Care by Bridging Biophilic Design, E-Commerce and Digital Health Technologies. Int. J. Environ. Res. Public Health.

[15] Bulaj G., Forero M., Huntsman D.D. (2025). Biophilic design, neuroarchitecture and therapeutic home environments: Harnessing medicinal properties of intentionally-designed spaces to enhance digital health outcomes. Front. Med..

[16] Al Khatib I., Samara F., Ndiaye M. (2024). A systematic review of the impact of therapeutical biophilic design on health and wellbeing of patients and care providers in healthcare services settings. Front. Built Environ..

[17] Browning W.D., Ryan C.O., Clancy J.O. (2024). 14 Patterns of Biophilic Design. Improving Health and Wellbeing in the Built Environment.

[18] Hung S.-H., Chang C.-Y. (2021). Health benefits of evidence-based biophilic-designed environments: A review. J. People Plants Environ..

[19] Kellert S.R., Heerwagen J., Mador M. (2008). Biophilic Design: The Theory, Science, and Practice of Bringing Buildings to Life.

[20] Tekin B.H., Corcoran R., Gutiérrez R.U. (2023). A Systematic Review and Conceptual Framework of Biophilic Design Parameters in Clinical Environments. Herd.

[21] Riva A., Rebecchi A., Capolongo S., Gola M. (2022). Can homes affect well-being? A scoping review among housing conditions, indoor environmental quality, and mental health outcomes. Int. J. Environ. Res. Public Health.

[22] Vardoulakis S., Giagloglou E., Steinle S., Davis A., Sleeuwenhoek A., Galea K.S., Dixon K., Crawford J.O. (2020). Indoor Exposure to Selected Air Pollutants in the Home Environment: A Systematic Review. Int. J. Environ. Res. Public Health.

[23] Allen J.G., Cedeno-Laurent J., Jones E., Luna M., Macnaughton P., Robinson S., Spengler J., Young A. (2019). Homes for Health: 36 Expert Tips to Make Your Home a Healthier Home.

[24] Osibona O., Solomon B.D., Fecht D. (2021). Lighting in the Home and Health: A Systematic Review. Int. J. Environ. Res. Public Health.

[25] Lin I., Glinsky J., Dean C., Graham P., Scrivener K. (2022). Effectiveness of home-based exercise for improving physical activity, quality of life and function in older adults after hospitalisation: A systematic review and meta-analysis. Clin. Rehabil..

[26] Barceló-Soler A., Morillo-Sarto H., Fernández-Martínez S., Monreal-Bartolomé A., Chambel M.J., Gardiner P., López-del-Hoyo Y., García-Campayo J., Pérez-Aranda A. (2023). A Systematic Review of the Adherence to Home-Practice Meditation Exercises in Patients with Chronic Pain. Int. J. Environ. Res. Public Health.

[27] Martino J., Pegg J., Frates E.P. (2017). The Connection Prescription: Using the Power of Social Interactions and the Deep Desire for Connectedness to Empower Health and Wellness. Am. J. Lifestyle Med..

[28] Holt-Lunstad J. (2022). Social Connection as a Public Health Issue: The Evidence and a Systemic Framework for Prioritizing the “Social” in Social Determinants of Health. Annu. Rev. Public Health.

[29] Hekmatmanesh A., Banaei M., Haghighi K.S., Najafi A. (2019). Bedroom design orientation and sleep electroencephalography signals. Acta Medica Int..

[30] Bedrov A., Gable S.L. (2023). Thriving together: The benefits of women’s social ties for physical, psychological and relationship health. Philos. Trans. R. Soc. B Biol. Sci..

[31] Sakallaris B.R., MacAllister L., Voss M., Smith K., Jonas W.B. (2015). Optimal healing environments. Glob. Adv. Health Med..

[32] Tekin B.H., Izmir Tunahan G., Disci Z.N., Ozer H.S. (2025). Biophilic Design in the Built Environment: Trends, Gaps and Future Directions. Buildings.

[33] Kellert S., Calabrese E. (2015). The Practice of Biophilic Design.

[34] McGee B., Park N.K., Portillo M., Bosch S., Swisher M. (2019). DIY Biophilia: Development of the Biophilic Interior Design Matrix as a design tool. J. Inter. Des..

[35] Browning W.D., Ryan C.O. (2020). Nature Inside: A Biophilic Design Guide.

[36] Kellert S.R. (2018). Nature by Design: The Practice of Biophilic Design.

[37] McSweeney J., Johnson S., Sherry S., Singleton J., Rainham D. (2021). Indoor nature exposure and influence on physiological stress markers. Int. J. Environ. Health Res..

[38] McSweeney J., Rainham D., Johnson S.A., Sherry S.B., Singleton J. (2015). Indoor nature exposure (INE): A health-promotion framework. Health Promot. Int..

[39] Rhee J.H., Schermer B., Han G., Park S.Y., Lee K.H. (2023). Effects of nature on restorative and cognitive benefits in indoor environment. Sci. Rep..

[40] Zhong W., Schröder T., Bekkering J. (2022). Biophilic design in architecture and its contributions to health, well-being, and sustainability: A critical review. Front. Archit. Res..

[41] Baquedano C., Olguí A., Contreras-Huerta L.S., Rosas F.E., Estarellas M. (2026). Your brain on nature: A scoping review of the neuroscience of nature exposure. Neurosci. Biobehav. Rev..

[42] Guidolin K., Jung F., Hunter S., Yan H., Englesakis M., Verderber S., Chadi S., Quereshy F. (2024). The Influence of Exposure to Nature on Inpatient Hospital Stays: A Scoping Review. Herd.

[43] Miola L., Boldrini A., Pazzaglia F. (2025). The healing power of nature. Biophilic design applied to healthcare facilities. Curr. Opin. Psychol..

[44] Suess C., Maddock J. (2025). Understanding the Influence of Window Views, Plantscapes, and Green Décor in Virtual Reality Hospital Rooms on Simulated Acute-Care Patients’ Stress Recovery and Relaxation Responses. HERD Health Environ. Res. Des. J..

[45] Ikei H., Song C., Sagasaki Y., Nozaki H., Miyazaki Y. (2025). Physiological effects of a small green space installed on the side of a clinic for outpatients with depression. Front. Environ. Health.

[46] Du Y., Xie M., Zou Y. (2026). Biophilic design in hospital environments: A rapid review of nature-integrated strategies and patient outcomes. Front. Public Health.

[47] Valentine C., Steffert T., Mitcheltree H., Steemers K. (2024). Architectural Neuroimmunology: A Pilot Study Examining the Impact of Biophilic Architectural Design on Neuroinflammation. Buildings.

[48] Andersen L., Corazon S.S., Stigsdotter U.K. (2021). Nature Exposure and Its Effects on Immune System Functioning: A Systematic Review. Int. J. Environ. Res. Public Health.

[49] Egorov A.I., Griffin S.M., Styles J.N., Kobylanski J., Klein J., Wickersham L., Ritter R., Sams E., Hudgens E.E., Wade T.J. (2024). Time outdoors and residential greenness are associated with reduced systemic inflammation and allostatic load. Environ. Pollut..

[50] Soininen L., Roslund M.I., Nurminen N., Puhakka R., Laitinen O.H., Hyöty H., Sinkkonen A., Cerrone D., Grönroos M., Hui N. (2022). Indoor green wall affects health-associated commensal skin microbiota and enhances immune regulation: A randomized trial among urban office workers. Sci. Rep..

[51] McGee B., Marshall-Baker A. (2015). Loving Nature From the Inside Out: A Biophilia Matrix Identification Strategy for Designers. HERD.

[52] Berto R., Barbiero G. (2017). The biophilic quality index. A tool to improve a building from “green” to restorative. Vis. Sustain..

[53] Salingaros N.A. (2019). The biophilic healing index predicts effects of the built environment on our wellbeing. J. Biourbanism.

[54] Wijesooriya N., Brambilla A., Markauskaite L. (2025). Biophilic Quality Matrix: A tool to evaluate the biophilic quality of a building during early design stage. Clean. Prod. Lett..

[55] Khalil M.H., Steemers K. (2026). The Neurobiophilia Index. Buildings.

[56] Bianchi E., Billington S.L. (2025). Nature view potential: Evaluating building occupant connection to nature. J. Build. Eng..

[57] Wolf K.L., Lam S.T., McKeen J.K., Richardson G.R.A., van den Bosch M., Bardekjian A.C. (2020). Urban Trees and Human Health: A Scoping Review. Int. J. Environ. Res. Public Health.

[58] Sander M., Klimesch A., Samaan L., Kühn S., Augustin J., Ascone L. (2025). Natural vs. built visual urban landscape elements around the home and their associations with mental and brain health of residents: A narrative review. J. Environ. Psychol..

[59] Nieuwenhuijsen M.J., Dadvand P., Márquez S., Bartoll X., Barboza E.P., Cirach M., Borrell C., Zijlema W.L. (2022). The evaluation of the 3-30-300 green space rule and mental health. Environ. Res..

[60] Raanaas R.K., Patil G.G., Hartig T. (2012). Health benefits of a view of nature through the window: A quasi-experimental study of patients in a residential rehabilitation center. Clin. Rehabil..

[61] Ulrich R.S. (1984). View through a window may influence recovery from surgery. Science.

[62] Elsadek M., Deshun Z., Liu B. (2024). High-rise window views: Evaluating the physiological and psychological impacts of green, blue, and built environments. Build. Environ..

[63] Farley K.M.J., Veitch J.A. (2001). A Room with a View: A Review of the Effects of Windows on Work and Well-Being.

[64] He S., Zhang W., Guan Y. (2025). The Impact of Building Windows on Occupant Well-Being: A Review Integrating Visual and Non-Visual Pathways with Multi-Objective Optimization. Buildings.

[65] Zhang J., Zhou S., Xia T., Yin Y., Wang X., Cheng Y., Mao Y., Zhao B. (2024). Residential greenspace exposure, particularly green window-views, is associated with improved sleep quality among older adults: Evidence from a high-density city. Build. Environ..

[66] Li H., Browning M.H.E.M., Bardhan M., Ying M., Zhang X., Cao Y., Zhang G. (2024). Nature connectedness connects the visibility of trees through windows and mental wellbeing: A study on the “3 visible trees” component of the 3-30-300 rule. Int. J. Environ. Health Res..

[67] Jonauskaite D., Mohr C. (2025). Do we feel colours? A systematic review of 128 years of psychological research linking colours and emotions. Psychon. Bull. Rev..

[68] Güneş E., Olguntürk N. (2020). Color-emotion associations in interiors. Color Res. Appl..

[69] Jiang Y., Li N., Yongga A., Yan W. (2022). Short-term effects of natural view and daylight from windows on thermal perception, health, and energy-saving potential. Build. Environ..

[70] West A., Simonsen S.A., Zielinski A., Cyril N., Schønsted M., Jennum P., Sander B., Iversen H.K. (2019). An exploratory investigation of the effect of naturalistic light on depression, anxiety, and cognitive outcomes in stroke patients during admission for rehabilitation: A randomized controlled trial. NeuroRehabilitation.

[71] West A., Simonsen S.A., Jennum P., Cyril Hansen N., Schønsted M., Zielinski A., Sander B., Iversen H.K. (2019). An exploratory investigation of the effect of naturalistic light on fatigue and subjective sleep quality in stroke patients admitted for rehabilitation: A randomized controlled trial. NeuroRehabilitation.

[72] Xiao H., Cai H., Li X. (2021). Non-visual effects of indoor light environment on humans: A review✰. Physiol. Behav..

[73] Awada M., Gerber B.B., Lucas G.M., Roll S.C. (2025). The impact of color correlated temperature and illuminance levels of office lighting on stress and cognitive restoration. J. Environ. Psychol..

[74] Kuijsters A., Redi J., de Ruyter B., Heynderickx I. (2015). Lighting to Make You Feel Better: Improving the Mood of Elderly People with Affective Ambiences. PLoS ONE.

[75] Ratcliffe E. (2021). Sound and Soundscape in Restorative Natural Environments: A Narrative Literature Review. Front. Psychol..

[76] Buxton R.T., Pearson A.L., Allou C., Fristrup K., Wittemyer G. (2021). A synthesis of health benefits of natural sounds and their distribution in national parks. Proc. Natl. Acad. Sci. USA.

[77] Song I., Baek K., Kim C., Song C. (2023). Effects of nature sounds on the attention and physiological and psychological relaxation. Urban For. Urban Green..

[78] Michels N., Hamers P. (2023). Nature Sounds for Stress Recovery and Healthy Eating: A Lab Experiment Differentiating Water and Bird Sound. Environ. Behav..

[79] Li Z., Zhang W., Cui J., Liu H., Liu H. (2024). Beneficial effects of short-term exposure to indoor biophilic environments on psychophysiological health: Evidence from electrophysiological activity and salivary metabolomics. Environ. Res..

[80] Lee M.-S., Lee J., Park B.-J., Miyazaki Y. (2015). Interaction with indoor plants may reduce psychological and physiological stress by suppressing autonomic nervous system activity in young adults: A randomized crossover study. J. Physiol. Anthropol..

[81] Aydogan A., Cerone R. (2021). Review of the effects of plants on indoor environments. Indoor Built Environ..

[82] Jiang S., Deng L., Luo H., Li X., Guo B., Jiang M., Jia Y., Ma J., Sun L., Huang Z. (2021). Effect of Fragrant Primula Flowers on Physiology and Psychology in Female College Students: An Empirical Study. Front. Psychol..

[83] Ikei H., Song C., Miyazaki Y. (2023). Physiological adjustment effect of visual stimulation by fresh rose flowers on sympathetic nervous activity. Front. Psychol..

[84] Shao Y., Zhou Z., Ding D., Cui Y., Wu X. (2024). Psychological Effects of a Living Wall System on Office Occupants: A Comparative Study Based on Physiological Responses. Buildings.

[85] Lipovac D., Burnard M.D. (2021). Effects of visual exposure to wood on human affective states, physiological arousal and cognitive performance: A systematic review of randomized trials. Indoor Built Environ..

[86] Kumpulainen S., Kilpiäinen M., Koski J., Pesola A.J. (2024). Wooden Interiors Improve Heart Rate Variability-Derived Psychophysiological Well-Being: An Acute Cross-Over Study. Environ. Behav..

[87] Zhang X., Lian Z., Wu Y. (2017). Human physiological responses to wooden indoor environment. Physiol. Behav..

[88] Ikei H., Jo H., Miyazaki Y. (2023). Physiological Effects of Visual Stimulation by a Japanese Low Wooden Table: A Crossover Field Experiment. Int. J. Environ. Res. Public Health.

[89] Ikei H., Song C., Miyazaki Y. (2017). Physiological Effects of Touching Wood. Int. J. Environ. Res. Public Health.

[90] Ikei H., Song C., Miyazaki Y. (2018). Physiological Effects of Touching the Wood of Hinoki Cypress (*Chamaecyparis obtusa*) with the Soles of the Feet. Int. J. Environ. Res. Public Health.

[91] Ikei H., Jo H., Miyazaki Y. (2024). Psychological and physiological effects of sole contact with oil-finished wood. J. Wood Sci..

[92] Özdil N., Ozcelik G., Supuren Menguc G., Jeon H.-Y. (2012). Sensorial Comfort of Textile Materials. Woven Fabrics.

[93] Bardalai R., Underwood J. (2022). SensAE—A Tool to Explore Material-Touch-Emotions. J. Text. Des. Res. Pract..

[94] Iosifyan M., Korolkova O. (2019). Emotions associated with different textures during touch. Conscious. Cogn..

[95] Etzi R., Spence C., Gallace A. (2014). Textures that we like to touch: An experimental study of aesthetic preferences for tactile stimuli. Conscious. Cogn..

[96] Taylor R.P. (2021). The Potential of Biophilic Fractal Designs to Promote Health and Performance: A Review of Experiments and Applications. Sustainability.

[97] Robles K.E., Roberts M., Viengkham C., Smith J.H., Rowland C., Moslehi S., Stadlober S., Lesjak A., Lesjak M., Taylor R.P. (2021). Aesthetics and psychological effects of fractal based design. Front. Psychol..

[98] Taylor R.P. (2006). Reduction of Physiological Stress Using Fractal Art and Architecture. Leonardo.

[99] Jo H., Song C., Miyazaki Y. (2019). Physiological Benefits of Viewing Nature: A Systematic Review of Indoor Experiments. Int. J. Environ. Res. Public Health.

[100] Cardillo E.R., Chatterjee A. (2025). Benefits of Nature Imagery and Visual Art in Healthcare Contexts: A View from Empirical Aesthetics. Buildings.

[101] Velarde M.D., Fry G., Tveit M. (2007). Health effects of viewing landscapes—Landscape types in environmental psychology. Urban For. Urban Green..

[102] Brown D.K., Barton J.L., Gladwell V.F. (2013). Viewing nature scenes positively affects recovery of autonomic function following acute-mental stress. Environ. Sci. Technol..

[103] Van den Berg A.E., Joye Y., Koole S.L. (2016). Why viewing nature is more fascinating and restorative than viewing buildings: A closer look at perceived complexity. Urban For. Urban Green..

[104] Ballew M.T., Omoto A.M. (2018). Absorption: How Nature Experiences Promote Awe and Other Positive Emotions. Ecopsychology.

[105] Jiang T., Hicks J.A., Yuan W., Yin Y., Needy L., Vess M. (2024). The unique nature and psychosocial implications of awe. Nat. Rev. Psychol..

[106] Monroy M., Keltner D. (2023). Awe as a Pathway to Mental and Physical Health. Perspect. Psychol. Sci..

[107] Ward Thompson C. (2011). Linking landscape and health: The recurring theme. Landsc. Urban Plan..

[108] Chalmin-Pui L.S., Griffiths A., Roe J., Heaton T., Cameron R. (2021). Why garden?—Attitudes and the perceived health benefits of home gardening. Cities.

[109] Soga M., Gaston K.J., Yamaura Y. (2017). Gardening is beneficial for health: A meta-analysis. Prev. Med. Rep..

[110] Wang F., Boros S. (2025). Effect of gardening activities on domains of health: A systematic review and meta-analysis. BMC Public Health.

[111] Howarth M., Brettle A., Hardman M., Maden M. (2020). What is the evidence for the impact of gardens and gardening on health and well-being: A scoping review and evidence-based logic model to guide healthcare strategy decision making on the use of gardening approaches as a social prescription. BMJ Open.

[112] Bavarsad N.H., Bagheri S., Kourosh-Arami M., Komaki A. (2023). Aromatherapy for the brain: Lavender’s healing effect on epilepsy, depression, anxiety, migraine, and Alzheimer’s disease: A review article. Heliyon.

[113] Sasannejad P., Saeedi M., Shoeibi A., Gorji A., Abbasi M., Foroughipour M. (2012). Lavender Essential Oil in the Treatment of Migraine Headache: A Placebo-Controlled Clinical Trial. Eur. Neurol..

[114] Lakhan S.E., Sheafer H., Tepper D. (2016). The Effectiveness of Aromatherapy in Reducing Pain: A Systematic Review and Meta-Analysis. Pain Res. Treat..

[115] Fang C.-S., Tu Y.-K., Chou F.-H., Fang C.-J., Chang S.-L. (2025). Effect of inhaled aromatherapy on sleep quality in critically ill patients: A systematic review and network meta-analysis. J. Clin. Nurs..

[116] Gong M., Dong H., Tang Y., Huang W., Lu F. (2020). Effects of aromatherapy on anxiety: A meta-analysis of randomized controlled trials. J. Affect. Disord..

[117] Farrow T. (2024). Constructing Health: How the Built Environment Enhances Your Mind’s Health.

[118] Valentine C. (2024). Architecture and public health: From harmful designs to healthy built environments. BMJ.

[119] Allen J.G., MacNaughton P., Laurent J.G.C., Flanigan S.S., Eitland E.S., Spengler J.D. (2015). Green Buildings and Health. Curr. Environ. Health Rep..

[120] Hodgson M. (2002). Indoor environmental exposures and symptoms. Environ. Health Perspect..

[121] Obeng A., Roh T., Moreno-Rangel A., Carrillo G. (2026). Evaluating the Effectiveness of Combined Indoor Air Quality Management and Asthma Education on Indoor Air Quality and Asthma Control in Adults. Atmosphere.

[122] Hansel N.N., Putcha N., Woo H., Peng R., Diette G.B., Fawzy A., Wise R.A., Romero K., Davis M.F., Rule A.M. (2022). Randomized clinical trial of air cleaners to improve indoor air quality and chronic obstructive pulmonary disease health: Results of the CLEAN AIR study. Am. J. Respir. Crit. Care Med..

[123] Torres G., Subbaiah R.T., Sood R.A., Leheste J.R. (2025). From air to mind: Unraveling the impact of indoor pollutants on psychiatric disorders. Front. Psychiatry.

[124] Menculini G., Cirimbilli F., Raspa V., Scopetta F., Cinesi G., Chieppa A.G., Cuzzucoli L., Moretti P., Balducci P.M., Attademo L. (2024). Insights into the Effect of Light Pollution on Mental Health: Focus on Affective Disorders—A Narrative Review. Brain Sci..

[125] Harmsen J.-F., Habets I., Biancolin A.D., Lesniewska A., Phillips N.E., Metz L., Sanchez-Avila J., Kotte M., Timmermans M., Hashim D. (2026). Natural daylight during office hours improves glucose control and whole-body substrate metabolism. Cell Metab..

[126] Yamagami Y., Obayashi K., Tai Y., Saeki K. (2023). Association between indoor noise level at night and objective/subjective sleep quality in the older population: A cross-sectional study of the HEIJO-KYO cohort. Sleep.

[127] Park S.H., Lee P.J. (2025). Effects of noise on health-related quality of life: The roles of outdoor noise, indoor noise, and noise sensitivity. J. Expo. Sci. Environ. Epidemiol..

[128] Posternack C., Kupchak P., Capriolo A.I., Katz B.J. (2023). Targeting the intrinsically photosensitive retinal ganglion cell to reduce headache pain and light sensitivity in migraine: A randomized double-blind trial. J. Clin. Neurosci..

[129] Soheilian M., Fischl G., Aries M. (2021). Smart lighting application for energy saving and user well-being in the residential environment. Sustainability.

[130] Blume C., Garbazza C., Spitschan M. (2019). Effects of light on human circadian rhythms, sleep and mood. Somnologie.

[131] Martin L.F., Patwardhan A.M., Jain S.V., Salloum M.M., Freeman J., Khanna R., Gannala P., Goel V., Jones-MacFarland F.N., Killgore W.D. (2021). Evaluation of green light exposure on headache frequency and quality of life in migraine patients: A preliminary one-way cross-over clinical trial. Cephalalgia.

[132] Lipton R.B., Melo-Carrillo A., Severs M., Reed M., Ashina S., Houle T., Burstein R. (2023). Narrow band green light effects on headache, photophobia, sleep, and anxiety among migraine patients: An open-label study conducted online using daily headache diary. Front. Neurol..

[133] Hou T.-W., Yang C.-C., Lai T.-H., Wu Y.-H., Yang C.-P. (2024). Light Therapy in Chronic Migraine. Curr. Pain Headache Rep..

[134] Martin L., Porreca F., Mata E.I., Salloum M., Goel V., Gunnala P., Killgore W.D.S., Jain S., Jones-MacFarland F.N., Khanna R. (2021). Green Light Exposure Improves Pain and Quality of Life in Fibromyalgia Patients: A Preliminary One-Way Crossover Clinical Trial. Pain Med..

[135] Shechter A., Quispe K.A., Mizhquiri Barbecho J.S., Slater C., Falzon L. (2020). Interventions to reduce short-wavelength (“blue”) light exposure at night and their effects on sleep: A systematic review and meta-analysis. Sleep Adv..

[136] Tähkämö L., Partonen T., Pesonen A.-K. (2019). Systematic review of light exposure impact on human circadian rhythm. Chronobiol. Int..

[137] Neophytou E., Manwell L.A., Eikelboom R. (2021). Effects of Excessive Screen Time on Neurodevelopment, Learning, Memory, Mental Health, and Neurodegeneration: A Scoping Review. Int. J. Ment. Health Addict..

[138] Nakshine V.S., Thute P., Khatib M.N., Sarkar B. (2022). Increased screen time as a cause of declining physical, psychological health, and sleep patterns: A literary review. Cureus.

[139] Anderl C., Hofer M.K., Chen F.S. (2024). Directly-measured smartphone screen time predicts well-being and feelings of social connectedness. J. Soc. Pers. Relatsh..

[140] Arundell L., Parker K., Timperio A., Salmon J., Veitch J. (2020). Home-based screen time behaviors amongst youth and their parents: Familial typologies and their modifiable correlates. BMC Public Health.

[141] Portt A.E., Orchard C., Chen H., Ge E., Lay C., Smith P.M. (2023). Migraine and air pollution: A systematic review. Headache J. Head Face Pain.

[142] Sabour S., Harzand-Jadidi S., Jafari-Khounigh A., Zarea Gavgani V., Sedaghat Z., Alavi N. (2024). The association between ambient air pollution and migraine: A systematic review. Environ. Monit. Assess..

[143] Dong H.-J., Ran P., Liao D.-Q., Chen X.-B., Chen G., Ou Y.-Q., Li Z.-H. (2024). Long-term exposure to air pollutants and new-onset migraine: A large prospective cohort study. Ecotoxicol. Environ. Saf..

[144] Lukina A.O., Burstein B., Szyszkowicz M. (2022). Urban air pollution and emergency department visits related to central nervous system diseases. PLoS ONE.

[145] Elser H., Kruse C.F.G., Schwartz B.S., Casey J.A. (2024). The Environment and Headache: A Narrative Review. Curr. Environ. Health Rep..

[146] Sjöstrand C., Savic I., Laudon-Meyer E., Hillert L., Lodin K., Waldenlind E. (2010). Migraine and Olfactory Stimuli. Curr. Pain Headache Rep..

[147] Cao B., Chen Y., McIntyre R.S. (2021). Comprehensive review of the current literature on impact of ambient air pollution and sleep quality. Sleep Med..

[148] Gao P. (2026). The human airborne exposome. Nat. Health.

[149] Ragab A., Hassieb M.M., Mohamed A.F. (2025). Exploring the impact of window design and ventilation strategies on air quality and thermal comfort in arid educational buildings. Sci. Rep..

[150] Li S., Liu Q., Ma M., Fang J., He L. (2025). Association between weather conditions and migraine: A systematic review and meta-analysis. J. Neurol..

[151] Tsang T.W., Mui K.W., Wong L.T. (2021). Investigation of thermal comfort in sleeping environment and its association with sleep quality. Build. Environ..

[152] Lan L., Tsuzuki K., Liu Y.F., Lian Z.W. (2017). Thermal environment and sleep quality: A review. Energy Build..

[153] Bai Z., Han Y., Zhuang D., Sun C. (2025). How do we create a healthier thermal environment for sleep? A review of sleep thermal comfort and sleep quality. Build. Environ..

[154] Münzel T., Molitor M., Kuntic M., Hahad O., Röösli M., Engelmann N., Basner M., Daiber A., Sørensen M. (2024). Transportation Noise Pollution and Cardiovascular Health. Circ. Res..

[155] Smith M.G., Cordoza M., Basner M. (2022). Environmental Noise and Effects on Sleep: An Update to the WHO Systematic Review and Meta-Analysis. Environ. Health Perspect..

[156] Polemiti E., Hese S., Schepanski K., Yuan J., Schumann G., Environmental Consortium (2024). How does the macroenvironment influence brain and behaviour—A review of current status and future perspectives. Mol. Psychiatry.

[157] Fancourt D., Ockelford A., Belai A. (2014). The psychoneuroimmunological effects of music: A systematic review and a new model. Brain Behav. Immun..

[158] Koelsch S., Boehlig A., Hohenadel M., Nitsche I., Bauer K., Sack U. (2016). The impact of acute stress on hormones and cytokines, and how their recovery is affected by music-evoked positive mood. Sci. Rep..

[159] Garza-Villarreal E.A., Pando V., Vuust P., Parsons C. (2017). Music-Induced Analgesia in Chronic Pain Conditions: A Systematic Review and Meta-Analysis. Pain Physician.

[160] Guduru R.K.R., Domeika A., Obcarskas L., Ylaite B. (2022). The Ergonomic Association between Shoulder, Neck/Head Disorders and Sedentary Activity: A Systematic Review. J. Healthc. Eng..

[161] Mazaheri-Tehrani S., Arefian M., Abhari A.P., Riahi R., Vahdatpour B., Mahdavi S.B., Kelishadi R. (2023). Sedentary behavior and neck pain in adults: A systematic review and meta-analysis. Prev. Med..

[162] Liu Y., Hu W., Kasal A., Erdil Y.Z. (2023). The State of the Art of Biomechanics Applied in Ergonomic Furniture Design. Appl. Sci..

[163] Rivilis I., Van Eerd D., Cullen K., Cole D.C., Irvin E., Tyson J., Mahood Q. (2008). Effectiveness of participatory ergonomic interventions on health outcomes: A systematic review. Appl. Ergon..

[164] Lietz J., Ulusoy N., Nienhaus A. (2020). Prevention of Musculoskeletal Diseases and Pain among Dental Professionals through Ergonomic Interventions: A Systematic Literature Review. Int. J. Environ. Res. Public Health.

[165] Thacher P., Onyper S., Tuthill J. (2017). 0373 de-Cluttering the Bedroom as a Possible Sleep Hygiene Step to Improve Sleep Quality. Sleep.

[166] Dion D., Sabri O., Guillard V. (2014). Home Sweet Messy Home: Managing Symbolic Pollution. J. Consum. Res..

[167] Rogers C.J., Hart D.R. (2021). Home and the extended-self: Exploring associations between clutter and wellbeing. J. Environ. Psychol..

[168] Yang Q., Wang J., Norbäck D. (2021). The home environment in a nationwide sample of multi-family buildings in Sweden: Associations with ocular, nasal, throat and dermal symptoms, headache, and fatigue among adults. Indoor Air.

[169] Zhang X., Norbäck D., Fan Q., Bai X., Li T., Zhang Y., Li B., Zhao Z., Huang C., Deng Q. (2019). Dampness and mold in homes across China: Associations with rhinitis, ocular, throat and dermal symptoms, headache and fatigue among adults. Indoor Air.

[170] Wang J., Janson C., Lindberg E., Holm M., Gislason T., Benediktsdóttir B., Johannessen A., Schlünssen V., Jogi R., Franklin K.A. (2020). Dampness and mold at home and at work and onset of insomnia symptoms, snoring and excessive daytime sleepiness. Environ. Int..

[171] Bondy S.C., Campbell A. (2018). Water Quality and Brain Function. Int. J. Environ. Res. Public Health.

[172] Wu J., Cao M., Tong D., Finkelstein Z., Hoek E.M.V. (2021). A critical review of point-of-use drinking water treatment in the United States. Npj Clean Water.

[173] Thompson R.C., Courtene-Jones W., Boucher J., Pahl S., Raubenheimer K., Koelmans A.A. (2024). Twenty years of microplastic pollution research—What have we learned?. Science.

[174] Chartres N., Cooper C.B., Bland G., Pelch K.E., Gandhi S.A., BakenRa A., Woodruff T.J. (2024). Effects of microplastic exposure on human digestive, reproductive, and respiratory health: A rapid systematic review. Environ. Sci. Technol..

[175] Nihart A.J., Garcia M.A., El Hayek E., Liu R., Olewine M., Kingston J.D., Castillo E.F., Gullapalli R.R., Howard T., Bleske B. (2025). Bioaccumulation of microplastics in decedent human brains. Nat. Med..

[176] Bora S.S., Gogoi R., Sharma M.R., Anshu, Borah M.P., Deka P., Bora J., Naorem R.S., Das J., Teli A.B. (2024). Microplastics and human health: Unveiling the gut microbiome disruption and chronic disease risks. Front. Cell. Infect. Microbiol..

[177] Yakovenko N., Pérez-Serrano L., Segur T., Hagelskjaer O., Margenat H., Le Roux G., Sonke J.E. (2025). Human exposure to PM10 microplastics in indoor air. PLoS ONE.

[178] Gettings S.M., Sharma R., Bourbia N. (2026). Micro- and nanoplastics in neurological dysfunction. Trends Neurosci..

[179] Luque-García L., Muxika-Legorburu J., Mendia-Berasategui O., Lertxundi A., García-Baquero G., Ibarluzea J. (2024). Green and blue space exposure and non-communicable disease related hospitalizations: A systematic review. Environ. Res..

[180] Liu Z., Chen X., Cui H., Ma Y., Gao N., Li X., Meng X., Lin H., Abudou H., Guo L. (2023). Green space exposure on depression and anxiety outcomes: A meta-analysis. Environ. Res..

[181] Yao W., Chen F., Wang S., Zhang X. (2021). Impact of Exposure to Natural and Built Environments on Positive and Negative Affect: A Systematic Review and Meta-Analysis. Front. Public Health.

[182] Yang T., Wang J., Huang J., Kelly F.J., Li G. (2023). Long-term Exposure to Multiple Ambient Air Pollutants and Association with Incident Depression and Anxiety. JAMA Psychiatry.

[183] Wolf K., Hoffmann B., Andersen Z.J., Atkinson R.W., Bauwelinck M., Bellander T., Brandt J., Brunekreef B., Cesaroni G., Chen J. (2021). Long-term exposure to low-level ambient air pollution and incidence of stroke and coronary heart disease: A pooled analysis of six European cohorts within the ELAPSE project. Lancet Planet. Health.

[184] Yuchi W., Sbihi H., Davies H., Tamburic L., Brauer M. (2020). Road proximity, air pollution, noise, green space and neurologic disease incidence: A population-based cohort study. Environ. Health.

[185] Münzel T., Kröller-Schön S., Oelze M., Gori T., Schmidt F.P., Steven S., Hahad O., Röösli M., Wunderli J.-M., Daiber A. (2020). Adverse Cardiovascular Effects of Traffic Noise with a Focus on Nighttime Noise and the New WHO Noise Guidelines. Annu. Rev. Public Health.

[186] Jensen H.A.R., Rasmussen B., Ekholm O. (2018). Neighbour and traffic noise annoyance: A nationwide study of associated mental health and perceived stress. Eur. J. Public Health.

[187] Hahad O., Foos P., Hübner J., Große-Dresselhaus C., Schmidt F.P., Ostad M.A., Kuntic M., Hobohm L., Keller K., Schmitt V.H. (2026). A randomized, double-blind, crossover study of acute low-level night-time road traffic noise: Effects on vascular function, sleep, and proteomic signatures in healthy adults. Cardiovasc. Res..

[188] Makram O.M., Pan A., Maddock J.E., Kash B.A. (2024). Nature and Mental Health in Urban Texas: A NatureScore-Based Study. Int. J. Environ. Res. Public Health.

[189] Makram O.M., Nwana N., Pan A.P., Nicolas C., Gullapelli R., Javed Z., Maddock J.E., Kash B., Nasir K. (2023). Nature Exposure and Cardiovascular Outcomes in a Large Metropolitan Area: The Role of Access to Nature. Circulation.

[190] Zheng Y., Lin T., Hamm N.A.S., Liu J., Zhou T., Geng H., Zhang J., Ye H., Zhang G., Wang X. (2024). Quantitative evaluation of urban green exposure and its impact on human health: A case study on the 3–30-300 green space rule. Sci. Total Environ..

[191] Khalil M.H. (2025). Green Environments for Sustainable Brains: Parameters Shaping Adaptive Neuroplasticity and Lifespan Neurosustainability—A Systematic Review and Future Directions. Int. J. Environ. Res. Public Health.

[192] Cardinali M., Beenackers M.A., van Timmeren A., Pottgiesser U. (2024). The relation between proximity to and characteristics of green spaces to physical activity and health: A multi-dimensional sensitivity analysis in four European cities. Environ. Res..

[193] Markevych I., Baumbach C., Helbich M., Burov A., Dimitrova D., Nieuwenhuijsen M.J., Dzhambov A.M. (2025). Does nature make us less lonely? Analysis in Bulgaria’s five largest cities. Health Place.

[194] Reid C.E., Rieves E.S., Carlson K. (2022). Perceptions of green space usage, abundance, and quality of green space were associated with better mental health during the COVID-19 pandemic among residents of Denver. PLoS ONE.

[195] Reklaitiene R., Grazuleviciene R., Dedele A., Virviciute D., Vensloviene J., Tamosiunas A., Baceviciene M., Luksiene D., Sapranaviciute-Zabazlajeva L., Radisauskas R. (2014). The relationship of green space, depressive symptoms and perceived general health in urban population. Scand. J. Public Health.

[196] Houlden V., Weich S., Porto de Albuquerque J., Jarvis S., Rees K. (2018). The relationship between greenspace and the mental wellbeing of adults: A systematic review. PLoS ONE.

[197] Chiang Y.-C., Li D. (2019). Metric or topological proximity? The associations among proximity to parks, the frequency of residents’ visits to parks, and perceived stress. Urban For. Urban Green..

[198] Makram O.M., Nwana N., Pan A., Nicolas J.C., Gullapelli R., Bose B., Sabharwal A., Chang J., Javed Z., Kash B. (2025). Interplay Between Residential Nature Exposure and Walkability and Their Association with Cardiovascular Health. JACC Adv..

[199] Nwana N., Javed Z., Jones S.L., Lee C., Maddock J.E., Al-Kindi S., Nasir K. (2024). Green Streets, Healthy Hearts: Exploring the Roles of Urban Nature and Walkability in Cardiovascular Health. Methodist. Debakey Cardiovasc. J..

[200] Yamada K., Hanazato M., Mizunuma N., Kondo N., Kondo K. (2025). Association of green space and climatic conditions with chronic pain and chronic widespread pain in older adults: A geo-epidemiologic cohort study. Urban For. Urban Green..

[201] Wang H., Gholami S., Xu W., Samavatekbatan A., Sleipness O., Tassinary L.G. (2024). Where and how to invest in greenspace for optimal health benefits: A systematic review of greenspace morphology and human health relationships. Lancet Planet. Health.

[202] Zock J.-P., Verheij R., Helbich M., Volker B., Spreeuwenberg P., Strak M., Janssen N.A.H., Dijst M., Groenewegen P. (2018). The impact of social capital, land use, air pollution and noise on individual morbidity in Dutch neighbourhoods. Environ. Int..

[203] Konijnendijk C.C. (2023). Evidence-based guidelines for greener, healthier, more resilient neighbourhoods: Introducing the 3–30–300 rule. J. For. Res..

[204] Richard A.A., Shea K. (2011). Delineation of self-care and associated concepts. J. Nurs. Scholarsh..

[205] World Health Organization (2022). WHO Guideline on Self-Care Interventions for Health and Well-Being, 2022 Revision.

[206] Probyn K., Bowers H., Mistry D., Caldwell F., Underwood M., Patel S., Sandhu H.K., Matharu M., Pincus T., On behalf of the CHESS team (2017). Non-pharmacological self-management for people living with migraine or tension-type headache: A systematic review including analysis of intervention components. BMJ Open.

[207] Short A.L. (2021). Enhancing migraine self-efficacy and reducing disability through a self-management program. J. Am. Assoc. Nurse Pract..

[208] Singh V., Kumar A., Ray S., Chakravarty K., Lall N., Joshi D. (2026). Non-pharmacological approaches for migraine management: A mini-review. Front. Pain Res..

[209] Duan S., Ren Z., Xia H., Wang Z., Zheng T., Liu Z. (2022). Association between sleep quality, migraine and migraine burden. Front. Neurol..

[210] Almansour N.A., Alsalamah S.S., Alsubaie R.S., Alshathri N.N., Alhedyan Y.A., Althekair’s F.Y. (2025). Association between migraine severity and sleep quality: A nationwide cross-sectional study. Front. Neurol..

[211] Lin Y.-K., Lin G.-Y., Lee J.-T., Lee M.-S., Tsai C.-K., Hsu Y.-W., Lin Y.-Z., Tsai Y.-C., Yang F.-C. (2016). Associations Between Sleep Quality and Migraine Frequency: A Cross-Sectional Case-Control Study. Medicine.

[212] Reina-Varona Á., Madroñero-Miguel B., Fierro-Marrero J., Paris-Alemany A., La Touche R. (2024). Efficacy of various exercise interventions for migraine treatment: A systematic review and network meta-analysis. Headache.

[213] Lemmens J., De Pauw J., Van Soom T., Michiels S., Versijpt J., Van Breda E., Castien R., De Hertogh W. (2019). The effect of aerobic exercise on the number of migraine days, duration and pain intensity in migraine: A systematic literature review and meta-analysis. J. Headache Pain.

[214] La Touche R., Fernandez Perez J.J., Proy Acosta A., Gonzalez Campodonico L., Martinez Garcia S., Adraos Juarez D., Serrano Garcia B., Angulo-Díaz-Parreño S., Cuenca-Martínez F., Suso-Martí L. (2020). Is aerobic exercise helpful in patients with migraine? A systematic review and meta-analysis. Scand. J. Med. Sci. Sports.

[215] Paudel P., Sah A. (2025). Efficacy of biofeedback for migraine: A systematic review and meta-analysis. Complement. Ther. Med..

[216] Treadwell J.R., Tsou A.Y., Rouse B., Ivlev I., Fricke J., Buse D.C., Powers S.W., Minen M., Szperka C.L., Mull N.K. (2025). Behavioral interventions for migraine prevention: A systematic review and meta-analysis. Headache J. Head Face Pain.

[217] Wells R.E., O’Connell N., Pierce C.R., Estave P., Penzien D.B., Loder E., Zeidan F., Houle T.T. (2021). Effectiveness of Mindfulness Meditation vs Headache Education for Adults With Migraine: A Randomized Clinical Trial. JAMA Intern. Med..

[218] García-Pérez-de-Sevilla G., González-de-la-Flor Á (2025). Impact of Fatty Acid Supplementation on Migraine Outcomes: A Systematic Review and Meta-analysis. Nutr. Rev..

[219] Talandashti M.K., Shahinfar H., Delgarm P., Jazayeri S. (2025). Effects of selected dietary supplements on migraine prophylaxis: A systematic review and dose–response meta-analysis of randomized controlled trials. Neurol. Sci..

[220] Dominguez L.J., Veronese N., Sabico S., Al-Daghri N.M., Barbagallo M. (2025). Magnesium and Migraine. Nutrients.

[221] Mugo C.W., Church E., Horniblow R.D., Mollan S.P., Botfield H., Hill L.J., Sinclair A.J., Grech O. (2025). Unravelling the gut-brain connection: A systematic review of migraine and the gut microbiome. J. Headache Pain.

[222] Demır Ü.F., Bozkurt O. (2020). Effects of Perceived Social Support, Depression and Anxiety Levels on Migraine. Noro Psikiyatr. Ars..

[223] D’Amico D., Grazzi L., Bussone G., Curone M., Di Fiore P., Usai S., Leonardi M., Giovannetti A.M., Schiavolin S., Raggi A. (2015). Are Depressive Symptomatology, Self-Efficacy, and Perceived Social Support Related to Disability and Quality of Life in Patients with Chronic Migraine Associated to Medication Overuse? Data From a Cross-Sectional Study. Headache J. Head Face Pain.

[224] Belam J., Harris G., Kernick D., Kline F., Lindley K., McWatt J., Mitchell A., Reinhold D. (2005). A qualitative study of migraine involving patient researchers. Br. J. Gen. Pract..

[225] Parlongue G., Cerdan E.V., Koenig J., Williams D.P. (2021). Smartphone based music intervention in the treatment of episodic migraine headaches—A pilot trial. Complement. Ther. Med..

[226] Koenig J., Oelkers-Ax R., Kaess M., Parzer P., Lenzen C., Hillecke T.K., Resch F. (2013). Specific Music Therapy Techniques in the Treatment of Primary Headache Disorders in Adolescents: A Randomized Attention-Placebo-Controlled Trial. J. Pain.

[227] Kindelan-Calvo P., Gil-Martínez A., Paris-Alemany A., Pardo-Montero J., Muñoz-García D., Angulo-Díaz-Parreño S., La Touche R. (2014). Effectiveness of Therapeutic Patient Education for Adults with Migraine. A Systematic Review and Meta-Analysis of Randomized Controlled Trials. Pain. Med..

[228] Rothrock J.F., Parada V.A., Sims C., Key K., Walters N.S., Zweifler R.M. (2006). The Impact of Intensive Patient Education on Clinical Outcome in a Clinic-Based Migraine Population. Headache J. Head Face Pain.

[229] Meise R., Schwarz A., Luedtke K. (2022). Effectiveness of Patient Education and Cognitive Behavioural Treatment as a Non-Pharmacological Intervention for Migraine in Adults—A Systematic Review. SN Compr. Clin. Med..

[230] Navalta J.W., McGinnis G.R., Malek E.M. (2024). Exercise in a natural environment increases program compliance in people with chronic migraine: A pilot cross-over randomized trial. J. Bodyw. Mov. Ther..

[231] Husak A.J., Bair M.J. (2020). Chronic pain and sleep disturbances: A pragmatic review of their relationships, comorbidities, and treatments. Pain Med..

[232] Whale K., Gooberman Hill R. (2022). The importance of sleep for people with chronic pain: Current insights and evidence. J. Bone Miner. Res. Plus.

[233] Goossens Z., Van Stallen A., Vermuyten J., Rice D., Runge N., Huysmans E., Vantilborgh T., Nijs J., Mairesse O., De Baets L. (2024). Day-to-day associations between pain intensity and sleep outcomes in an adult chronic musculoskeletal pain population: A systematic review. Sleep Med. Rev..

[234] Jain S.V., Panjeton G.D., Martins Y.C. (2024). Relationship Between Sleep Disturbances and Chronic Pain: A Narrative Review. Clin. Pract..

[235] García-Correa H.R., Sánchez-Montoya L.J., Daza-Arana J.E., Ordoñez-Mora L.T. (2021). Aerobic physical exercise for pain intensity, aerobic capacity, and quality of life in patients with chronic pain: A systematic review and meta-analysis. J. Phys. Act. Health.

[236] Ferro Moura Franco K., Lenoir D., dos Santos Franco Y.R., Jandre Reis F.J., Nunes Cabral C.M., Meeus M. (2021). Prescription of exercises for the treatment of chronic pain along the continuum of nociplastic pain: A systematic review with meta-analysis. Eur. J. Pain.

[237] Zhang Y.H., Hu H.Y., Xiong Y.C., Peng C., Hu L., Kong Y.Z., Wang Y.L., Guo J.B., Bi S., Li T.S. (2021). Exercise for Neuropathic Pain: A Systematic Review and Expert Consensus. Front. Med..

[238] Anheyer D., Haller H., Barth J., Lauche R., Dobos G., Cramer H. (2017). Mindfulness-based stress reduction for treating low back pain: A systematic review and meta-analysis. Ann. Intern. Med..

[239] Paschali M., Lazaridou A., Sadora J., Papianou L., Garland E.L., Zgierska A.E., Edwards R.R. (2024). Mindfulness-based interventions for chronic low back pain: A systematic review and meta-analysis. Clin. J. Pain.

[240] Lin T.-H., Tam K.-W., Yang Y.-L., Liou T.-H., Hsu T.-H., Rau C.-L. (2022). Meditation-based therapy for chronic low back pain management: A systematic review and meta-analysis of randomized controlled trials. Pain Med..

[241] Brain K., Burrows T.L., Rollo M.E., Chai L.K., Clarke E.D., Hayes C., Hodson F.J., Collins C.E. (2019). A systematic review and meta-analysis of nutrition interventions for chronic noncancer pain. J. Hum. Nutr. Diet..

[242] Field R., Pourkazemi F., Turton J., Rooney K. (2020). Dietary Interventions Are Beneficial for Patients with Chronic Pain: A Systematic Review with Meta-Analysis. Pain Med..

[243] Hajimirzaei P., Eyni H., Razmgir M., Abolfazli S., Pirzadeh S., Ahmadi Tabatabaei F.S., Vasigh A., Yazdanian N., Ramezani F., Janzadeh A. (2025). The analgesic effect of curcumin and nano-curcumin in clinical and preclinical studies: A systematic review and meta-analysis. Naunyn-Schmiedeberg Arch. Pharmacol..

[244] Yong W.C., Sanguankeo A., Upala S. (2017). Effect of vitamin D supplementation in chronic widespread pain: A systematic review and meta-analysis. Clin. Rheumatol..

[245] Rinaudo C.M., Van de Velde M., Steyaert A., Mouraux A. (2025). Navigating the biopsychosocial landscape: A systematic review on the association between social support and chronic pain. PLoS ONE.

[246] Che X., Cash R., Ng S.K., Fitzgerald P., Fitzgibbon B.M. (2018). A Systematic Review of the Processes Underlying the Main and the Buffering Effect of Social Support on the Experience of Pain. Clin. J. Pain.

[247] Gong C., Shan H., Sun Y., Zheng J., Zhu C., Zhong W., Guo J., Chen B. (2024). Social support as a key factor in chronic pain management programs: A scoping review. Curr. Psychol..

[248] Gonder M.E., Orr W.N., Khan T.W. (2022). The impact of isolation during COVID-19 on chronic musculoskeletal pain in the geriatric population: A narrative review. Pain Physician.

[249] McMurtry M., Viswanath O., Cernich M., Strand N., Freeman J., Townsend C., Kaye A.D., Cornett E.M., Wie C. (2020). The Impact of the Quantity and Quality of Social Support on Patients with Chronic Pain. Curr. Pain Headache Rep..

[250] Franqueiro A.R., Yoon J., Crago M.A., Curiel M., Wilson J.M. (2023). The Interconnection Between Social Support and Emotional Distress Among Individuals with Chronic Pain: A Narrative Review. Psychol. Res. Behav. Manag..

[251] Hsu H.F., Chen K.M., Belcastro F. (2022). The effect of music interventions on chronic pain experienced by older adults: A systematic review. J. Nurs. Scholarsh..

[252] Lepri B., Romani D., Storari L., Barbari V. (2023). Effectiveness of Pain Neuroscience Education in Patients with Chronic Musculoskeletal Pain and Central Sensitization: A Systematic Review. Int. J. Environ. Res. Public Health.

[253] Suso-Martí L., Cuenca-Martínez F., Alba-Quesada P., Muñoz-Alarcos V., Herranz-Gómez A., Varangot-Reille C., Domínguez-Navarro F., Casaña J. (2022). Effectiveness of pain neuroscience education in patients with fibromyalgia: A systematic review and meta-analysis. Pain Med..

[254] Marris D., Theophanous K., Cabezon P., Dunlap Z., Donaldson M. (2021). The impact of combining pain education strategies with physical therapy interventions for patients with chronic pain: A systematic review and meta-analysis of randomized controlled trials. Physiother. Theory Pract..

[255] Lovell M.R., Luckett T., Boyle F.M., Phillips J., Agar M., Davidson P.M. (2014). Patient education, coaching, and self-management for cancer pain. J. Clin. Oncol..

[256] Smith A., Wyles K.J., Hernandez S.M., Clarke S., Schofield P., Hughes S.W. (2025). Harnessing the therapeutic effects of nature for chronic Pain: A role for immersive virtual reality? A narrative review. Eur. J. Pain.

[257] Stanhope J., Breed M.F., Weinstein P. (2020). Exposure to greenspaces could reduce the high global burden of pain. Environ. Res..

[258] Wells N.M., Rollings K.A., Ong A.D., Reid M.C. (2019). Nearby Nature Buffers the Pain Catastrophizing–Pain Intensity Relation Among Urban Residents With Chronic Pain. Front. Built Environ..

[259] O’Callaghan V.S., Couvy-Duchesne B., Strike L.T., McMahon K.L., Byrne E.M., Wright M.J. (2021). A meta-analysis of the relationship between subjective sleep and depressive symptoms in adolescence. Sleep Med..

[260] Li X.-L., Wei J., Zhang X., Meng Z., Zhu W. (2023). Relationship between night-sleep duration and risk for depression among middle-aged and older people: A dose–response meta-analysis. Front. Physiol..

[261] Zhang M.-M., Ma Y., Du L.-T., Wang K., Li Z., Zhu W., Sun Y.-H., Lu L., Bao Y.-P., Li S.-X. (2022). Sleep disorders and non-sleep circadian disorders predict depression: A systematic review and meta-analysis of longitudinal studies. Neurosci. Biobehav. Rev..

[262] Pearce M., Garcia L., Abbas A., Strain T., Schuch F.B., Golubic R., Kelly P., Khan S., Utukuri M., Laird Y. (2022). Association between physical activity and risk of depression: A systematic review and meta-analysis. JAMA Psychiatry.

[263] Recchia F., Bernal J.D., Fong D.Y., Wong S.H., Chung P.-K., Chan D.K., Capio C.M., Yu C.C., Wong S.W., Sit C.H. (2023). Physical activity interventions to alleviate depressive symptoms in children and adolescents: A systematic review and meta-analysis. JAMA Pediatr..

[264] Heissel A., Heinen D., Brokmeier L.L., Skarabis N., Kangas M., Vancampfort D., Stubbs B., Firth J., Ward P.B., Rosenbaum S. (2023). Exercise as medicine for depressive symptoms? A systematic review and meta-analysis with meta-regression. Br. J. Sports Med..

[265] Noetel M., Sanders T., Gallardo-Gómez D., Taylor P., del Pozo Cruz B., Van Den Hoek D., Smith J.J., Mahoney J., Spathis J., Moresi M. (2024). Effect of exercise for depression: Systematic review and network meta-analysis of randomised controlled trials. BMJ.

[266] Reangsing C., Rittiwong T., Schneider J.K. (2021). Effects of mindfulness meditation interventions on depression in older adults: A meta-analysis. Aging Ment. Health.

[267] Reangsing C., Lauderman C., Schneider J.K. (2022). Effects of mindfulness meditation intervention on depressive symptoms in emerging adults: A systematic review and meta-analysis. J. Integr. Complement. Med..

[268] Fu Y., Song Y., Li Y., Sanchez-Vidana D.I., Zhang J.J., Lau W.K.W., Tan D.G.H., Ngai S.P.C., Lau B.W.-M. (2024). The effect of mindfulness meditation on depressive symptoms during the COVID-19 pandemic: A systematic review and meta-analysis. Sci. Rep..

[269] Wu P.-Y., Chen K.-M., Belcastro F. (2020). Dietary patterns and depression risk in older adults: Systematic review and meta-analysis. Nutr. Rev..

[270] Lassale C., Batty G.D., Baghdadli A., Jacka F., Sánchez-Villegas A., Kivimäki M., Akbaraly T. (2019). Healthy dietary indices and risk of depressive outcomes: Asystematic review and meta-analysis of observational studies. Mol. Psychiatry.

[271] Srifuengfung M., Srifuengfung S., Pummangura C., Pattanaseri K., Oon-Arom A., Srisurapanont M. (2023). Efficacy and acceptability of vitamin D supplements for depressed patients: A systematic review and meta-analysis of randomized controlled trials. Nutrition.

[272] Ng Q.X., Venkatanarayanan N., Ho C.Y. (2017). Clinical use of Hypericum perforatum (St John’s wort) in depression: A meta-analysis. J. Affect. Disord..

[273] Urata M., Sakurai H., Ueno F., Maruki T., Tada T., Uchida T., Matsumoto Y., Murao M., Tomita M., Baba H. (2025). Efficacy of Pharmacological Interventions in Milder Depression: A Systematic Review and Meta-Analysis. Neuropsychopharmacol. Rep..

[274] Gariépy G., Honkaniemi H., Quesnel-Vallée A. (2016). Social support and protection from depression: Systematic review of current findings in Western countries. Br. J. Psychiatry.

[275] Rueger S.Y., Malecki C.K., Pyun Y., Aycock C., Coyle S. (2016). A meta-analytic review of the association between perceived social support and depression in childhood and adolescence. Psychol. Bull..

[276] George L.K., Blazer D.G., Hughes D.C., Fowler N. (1989). Social support and the outcome of major depression. Br. J. Psychiatry.

[277] Lee S.H., Lee H., Yu S. (2022). Effectiveness of Social Support for Community-Dwelling Elderly with Depression: A Systematic Review and Meta-Analysis. Healthcare.

[278] Tang Q., Huang Z., Zhou H., Ye P. (2020). Effects of music therapy on depression: A meta-analysis of randomized controlled trials. PLoS ONE.

[279] Dhippayom T., Saensook T., Promkhatja N., Teaktong T., Chaiyakunapruk N., Devine B. (2022). Comparative effects of music interventions on depression in older adults: A systematic review and network meta-analysis. eClinicalMedicine.

[280] Zhao K., Bai Z.G., Bo A., Chi I. (2016). A systematic review and meta-analysis of music therapy for the older adults with depression. Int. J. Geriatr. Psychiatry.

[281] Wang M., Wu J., Yan H. (2023). Effect of music therapy on older adults with depression: A systematic review and meta-analysis. Complement. Ther. Clin. Pract..

[282] Katsuki F., Watanabe N., Yamada A., Hasegawa T. (2022). Effectiveness of family psychoeducation for major depressive disorder: Systematic review and meta-analysis. BJPsych Open.

[283] Bevan Jones R., Thapar A., Stone Z., Thapar A., Jones I., Smith D., Simpson S. (2018). Psychoeducational interventions in adolescent depression: A systematic review. Patient Educ. Couns..

[284] Cuijpers P. (1998). A psychoeducational approach to the treatment of depression: A meta-analysis of lewinsohn’s “coping with depression” course. Behav. Ther..

[285] Roberts H., van Lissa C., Hagedoorn P., Kellar I., Helbich M. (2019). The effect of short-term exposure to the natural environment on depressive mood: A systematic review and meta-analysis. Environ. Res..

[286] Siah C.J.R., Goh Y.S., Lee J., Poon S.N., Ow Yong J.Q.Y., Tam W.S.W. (2023). The effects of forest bathing on psychological well-being: A systematic review and meta-analysis. Int. J. Ment. Health Nurs..

[287] Chen H., Meng Z., Luo J. (2025). Is forest bathing a panacea for mental health problems? A narrative review. Front. Public Health.

[288] Cox R.C., Olatunji B.O. (2016). A systematic review of sleep disturbance in anxiety and related disorders. J. Anxiety Disord..

[289] Pires G.N., Bezerra A.G., Tufik S., Andersen M.L. (2016). Effects of acute sleep deprivation on state anxiety levels: A systematic review and meta-analysis. Sleep Med..

[290] Peng A., Ji S., Lai W., Hu D., Wang M., Zhao X., Chen L. (2024). The bidirectional relationship between sleep disturbance and anxiety: Sleep disturbance is a stronger predictor of anxiety. Sleep Med..

[291] Singh B., Olds T., Curtis R., Dumuid D., Virgara R., Watson A., Szeto K., O’Connor E., Ferguson T., Eglitis E. (2023). Effectiveness of physical activity interventions for improving depression, anxiety and distress: An overview of systematic reviews. Br. J. Sports Med..

[292] Carter T., Pascoe M., Bastounis A., Morres I.D., Callaghan P., Parker A.G. (2021). The effect of physical activity on anxiety in children and young people: A systematic review and meta-analysis. J. Affect. Disord..

[293] McDowell C.P., Dishman R.K., Gordon B.R., Herring M.P. (2019). Physical Activity and Anxiety: A Systematic Review and Meta-analysis of Prospective Cohort Studies. Am. J. Prev. Med..

[294] Zhou X., Guo J., Lu G., Chen C., Xie Z., Liu J., Zhang C. (2020). Effects of mindfulness-based stress reduction on anxiety symptoms in young people: A systematic review and meta-analysis. Psychiatry Res..

[295] Montero-Marin J., Garcia-Campayo J., Pérez-Yus M.C., Zabaleta-del-Olmo E., Cuijpers P. (2019). Meditation techniques v. relaxation therapies when treating anxiety: A meta-analytic review. Psychol. Med..

[296] Kim H.-S., Kim E.J. (2018). Effects of Relaxation Therapy on Anxiety Disorders: A Systematic Review and Meta-analysis. Arch. Psychiatr. Nurs..

[297] Manzoni G.M., Pagnini F., Castelnuovo G., Molinari E. (2008). Relaxation training for anxiety: A ten-years systematic review with meta-analysis. BMC Psychiatry.

[298] Lee A.R.Y.B., Tariq A., Lau G., Tok N.W.K., Tam W.W.S., Ho C.S.H. (2022). Vitamin E, Alpha-Tocopherol, and Its Effects on Depression and Anxiety: A Systematic Review and Meta-Analysis. Nutrients.

[299] Moshfeghinia R., Sanaei E., Mostafavi S., Assadian K., Sanaei A., Ayano G. (2024). The effects of L-theanine supplementation on the outcomes of patients with mental disorders: A systematic review. BMC Psychiatry.

[300] Rawji A., Peltier M.R., Mourtzanakis K., Awan S., Rana J., Pothen N.J., Afzal S. (2024). Examining the Effects of Supplemental Magnesium on Self-Reported Anxiety and Sleep Quality: A Systematic Review. Cureus.

[301] Dour H.J., Wiley J.F., Roy-Byrne P., Stein M.B., Sullivan G., Sherbourne C.D., Bystritsky A., Rose R.D., Craske M.G. (2014). Perceived social support mediates anxiety and depressive symptom changes following primary care intervention. Depress. Anxiety.

[302] Panayiotou G., Karekla M. (2013). Perceived social support helps, but does not buffer the negative impact of anxiety disorders on quality of life and perceived stress. Soc. Psychiatry Psychiatr. Epidemiol..

[303] Navarro-Nolasco D.A., Chi-Castañeda D., López-Meraz M.L., Beltran-Parrazal L., Morgado-Valle C. (2025). The medial prefrontal cortex as a proposed regulatory structure in the relationship between anxiety and perceived social support: A review. BMC Psychol..

[304] Harney C., Johnson J., Bailes F., Havelka J. (2023). Is music listening an effective intervention for reducing anxiety? A systematic review and meta-analysis of controlled studies. Music. Sci..

[305] Lu G., Jia R., Liang D., Yu J., Wu Z., Chen C. (2021). Effects of music therapy on anxiety: A meta-analysis of randomized controlled trials. Psychiatry Res..

[306] de Witte M., Aalbers S., Vink A., Friederichs S., Knapen A., Pelgrim T., Lampit A., Baker F.A., van Hooren S. (2025). Music therapy for the treatment of anxiety: A systematic review with multilevel meta-analyses. eClinicalMedicine.

[307] Moreno-Peral P., Conejo-Ceron S., Rubio-Valera M., Fernandez A., Navas-Campaña D., Rodriguez-Morejon A., Motrico E., Rigabert A., de Dios Luna J., Martin-Perez C. (2017). Effectiveness of psychological and/or educational interventions in the prevention of anxiety: A systematic review, meta-analysis, and meta-regression. JAMA Psychiatry.

[308] Rodrigues F., Bartolo A., Pacheco E., Pereira A., Silva C., Oliveira C. (2018). Psycho-education for anxiety disorders in adults: A systematic review of its effectiveness. J. Forensic Psychol..

[309] Zhang X., Zhang Y., Yun J., Yao W. (2023). A systematic review of the anxiety-alleviation benefits of exposure to the natural environment. Rev. Environ. Health.

[310] Wang X., Feng B., Wang J. (2025). Green spaces, blue spaces and human health: An updated umbrella review of epidemiological meta-analyses. Front. Public Health.

[311] Stepanski E.J., Wyatt J.K. (2003). Use of sleep hygiene in the treatment of insomnia. Sleep Med. Rev..

[312] Varadharasu S., Das N. (2024). Sleep hygiene efficacy on quality of sleep and mental ability among insomniac patients. J. Fam. Med. Prim. Care.

[313] Riedel A., Benz F., Deibert P., Barsch F., Frase L., Johann A.F., Riemann D., Feige B. (2024). The effect of physical exercise interventions on insomnia: A systematic review and meta-analysis. Sleep Med. Rev..

[314] Xie Y., Liu S., Chen X.-J., Yu H.-H., Yang Y., Wang W. (2021). Effects of Exercise on Sleep Quality and Insomnia in Adults: A Systematic Review and Meta-Analysis of Randomized Controlled Trials. Front. Psychiatry.

[315] Tian C., Wei Y., Xu M., Liu J., Tong B., Ning J., Wang Y., Wang Y., Estill J., Ge L. (2024). The effects of exercise on insomnia disorders: An umbrella review and network meta-analysis. Sleep Med..

[316] Bahalayothin P., Nagaviroj K., Anothaisintawee T. (2025). Impact of different types of physical exercise on sleep quality in older population with insomnia: A systematic review and network meta-analysis of randomised controlled trials. Fam. Med. Community Health.

[317] Chen T.-L., Chang S.-C., Hsieh H.-F., Huang C.-Y., Chuang J.-H., Wang H.-H. (2020). Effects of mindfulness-based stress reduction on sleep quality and mental health for insomnia patients: A meta-analysis. J. Psychosom. Res..

[318] Li H., Qin W., Li N., Feng S., Wang J., Zhang Y., Wang T., Wang C., Cai X., Sun W. (2023). Effect of mindfulness on anxiety and depression in insomnia patients: A systematic review and meta-analysis. Front. Psychiatry.

[319] Jerath R., Beveridge C., Barnes V.A. (2019). Self-Regulation of Breathing as an Adjunctive Treatment of Insomnia. Front. Psychiatry.

[320] Musgrave R.H., Nowakowski S., Watermeyer T.J., Arentson-Lantz E.J., Elder G.J. (2025). Dietary interventions to support and improve sleep disturbances and insomnia disorder in menopause: From bench to bedside. Post Reprod. Health.

[321] Polasek D., Santhi N., Alfonso-Miller P., Walshe I.H., Haskell-Ramsay C.F., Elder G.J. (2024). Nutritional interventions in treating menopause-related sleep disturbances: A systematic review. Nutr. Rev..

[322] Polianovskaia A., Jonelis M., Cheung J. (2024). The impact of plant-rich diets on sleep: A mini-review. Front. Nutr..

[323] Arab A., Karimi E., Garaulet M., Scheer F.A.J.L. (2024). Dietary patterns and insomnia symptoms: A systematic review and meta-analysis. Sleep Med. Rev..

[324] Seo S., Mattos M.K. (2024). The relationship between social support and sleep quality in older adults: A review of the evidence. Arch. Gerontol. Geriatr..

[325] Maratia F., Bacaro V., Crocetti E. (2023). Sleep Is a Family Affair: A Systematic Review and Meta-Analysis of Longitudinal Studies on the Interplay between Adolescents’ Sleep and Family Factors. Int. J. Environ. Res. Public Health.

[326] Gordon A.M., Carrillo B., Barnes C.M. (2021). Sleep and social relationships in healthy populations: A systematic review. Sleep Med. Rev..

[327] Feng F., Zhang Y., Hou J., Cai J., Jiang Q., Li X., Zhao Q., Li B.-A. (2018). Can music improve sleep quality in adults with primary insomnia? A systematic review and network meta-analysis. Int. J. Nurs. Stud..

[328] Tang Y.W., Teoh S.L., Yeo J.H.H., Ngim C.F., Lai N.M., Durrant S.J., Lee S.W.H. (2022). Music-based Intervention for Improving Sleep Quality of Adults without Sleep Disorder: A Systematic Review and Meta-analysis. Behav. Sleep Med..

[329] Jespersen K.V., Hansen M.H., Vuust P. (2023). The effect of music on sleep in hospitalized patients: A systematic review and meta-analysis. Sleep Health.

[330] Chen C.-T., Tung H.-H., Fang C.-J., Wang J.-L., Ko N.-Y., Chang Y.-J., Chen Y.-C. (2021). Effect of music therapy on improving sleep quality in older adults: A systematic review and meta-analysis. J. Am. Geriatr. Soc..

[331] Chung K.-F., Lee C.-T., Yeung W.-F., Chan M.-S., Chung E.W.-Y., Lin W.-L. (2017). Sleep hygiene education as a treatment of insomnia: A systematic review and meta-analysis. Fam. Pract..

[332] Ruan J.Y., Liu Q., Chung K.F., Ho K.Y., Yeung W.F. (2025). Effects of sleep hygiene education for insomnia: A systematic review and meta-analysis. Sleep Med. Rev..

[333] Astell-Burt T., Feng X., Kolt G.S. (2013). Does access to neighbourhood green space promote a healthy duration of sleep? Novel findings from a cross-sectional study of 259 319 Australians. BMJ Open.

[334] Shin J.C., Parab K.V., An R., Grigsby-Toussaint D.S. (2020). Greenspace exposure and sleep: A systematic review. Environ. Res..

[335] Johnson B.S., Malecki K.M., Peppard P.E., Beyer K.M.M. (2018). Exposure to neighborhood green space and sleep: Evidence from the Survey of the Health of Wisconsin. Sleep Health.

[336] Graham L.T., Gosling S.D., Travis C.K. (2015). The psychology of home environments: A call for research on residential space. Perspect. Psychol. Sci..

[337] Rantala E., Vanhatalo S., Tilles-Tirkkonen T., Kanerva M., Hansen P.G., Kolehmainen M., Männikkö R., Lindström J., Pihlajamäki J., Poutanen K. (2021). Choice Architecture Cueing to Healthier Dietary Choices and Physical Activity at the Workplace: Implementation and Feasibility Evaluation. Nutrients.

[338] Landais L.L., Damman O.C., Schoonmade L.J., Timmermans D.R.M., Verhagen E., Jelsma J.G.M. (2020). Choice architecture interventions to change physical activity and sedentary behavior: A systematic review of effects on intention, behavior and health outcomes during and after intervention. Int. J. Behav. Nutr. Phys. Act..

[339] Forberger S., Wichmann F., Comito C.N. (2022). Nudges used to promote physical activity and to reduce sedentary behaviour in the workplace: Results of a scoping review. Prev. Med..

[340] Baranwal N., Yu P.K., Siegel N.S. (2023). Sleep physiology, pathophysiology, and sleep hygiene. Prog. Cardiovasc. Dis..

[341] Irish L.A., Kline C.E., Gunn H.E., Buysse D.J., Hall M.H. (2015). The role of sleep hygiene in promoting public health: A review of empirical evidence. Sleep Med. Rev..

[342] Desaulniers J., Desjardins S., Lapierre S., Desgagné A. (2018). Sleep Environment and Insomnia in Elderly Persons Living at Home. J. Aging Res..

[343] Grote V., Frühwirth M., Lackner H.K., Goswami N., Köstenberger M., Likar R., Moser M. (2021). Cardiorespiratory Interaction and Autonomic Sleep Quality Improve during Sleep in Beds Made from Pinus cembra (Stone Pine) Solid Wood. Int. J. Environ. Res. Public Health.

[344] Morita E., Yanagisawa M., Ishihara A., Matsumoto S., Suzuki C., Ikeda Y., Ishitsuka M., Hori D., Doki S., Oi Y. (2020). Association of wood use in bedrooms with comfort and sleep among workers in Japan: A cross-sectional analysis of the SLeep Epidemiology Project at the University of Tsukuba (SLEPT) study. J. Wood Sci..

[345] Radwan A., Fess P., James D., Murphy J., Myers J., Rooney M., Taylor J., Torii A. (2015). Effect of different mattress designs on promoting sleep quality, pain reduction, and spinal alignment in adults with or without back pain; systematic review of controlled trials. Sleep Health.

[346] Bellini S., Miola L., Sperduti A., Caccaro A., Pinton E., Graffeo M., Pazzaglia F. (2025). Green design in living and bedroom spaces: Exploring environmental restorativeness and affective qualities of spaces. Front. Psychol..

[347] Lin X., Ji T., Guo R., Guo C., Gong P., Lan L. (2026). Association of bedroom particulate matter, sleep quality and next-day physical performance. Sci. Rep..

[348] Ellsworth-Krebs K., Reid L., Hunter C.J. (2019). Integrated framework of home comfort: Relaxation, companionship and control. Build. Res. Inf..

[349] Jutzi C.A., Möller J., Hansen J., Klackl J., Jonas E. (2025). Psychological needs in the built environment. J. Environ. Psychol..

[350] Guan H., Zhang X., Dong J., Shu R., Hu S., Tong Z. (2025). Biophilic environment with auditory-olfactory stimuli contributes to psychophysiological restoration from stress. Build. Environ..

[351] Woldeamanuel Y.W., Oliveira A.B.D. (2022). What is the efficacy of aerobic exercise versus strength training in the treatment of migraine? A systematic review and network meta-analysis of clinical trials. J. Headache Pain.

[352] Wanderley D., Valença M.M., de Souza Costa Neto J.J., Martins J.V., Raposo M.C.F., de Oliveira D.A. (2020). Contract-relax technique compared to static stretching in treating migraine in women: A randomized pilot trial. J. Bodyw. Mov. Ther..

[353] La Touche R., de Oliveira A.B., Paris-Alemany A., Reina-Varona Á (2024). Incorporating Therapeutic Education and Exercise in Migraine Management: A Biobehavioral Approach. J. Clin. Med..

[354] Ha W.-S., Chu M.K. (2024). Advances in Exercise in the Clinical Trials of Migraine: A Scoping Review. Curr. Pain Headache Rep..

[355] Bustamante J.G., Ramos V.J., Pastor-Cisneros R., Denche-Zamorano Á (2025). Migraine and physical activity: Prevalence, depression, and anxiety—A cross-sectional study. Sport Sci. Health.

[356] Lui J.Z., Young N.P., Ebbert J.O., Rosedahl J.K., Philpot L.M. (2020). Loneliness and Migraine Self-Management: A Cross-Sectional Assessment. J. Prim. Care Community Health.

[357] Colenberg S., Appel-Meulenbroek R., Romero Herrera N., Keyson D. (2024). Interior designers’ strategies for creating social office space. Ergonomics.

[358] Goldstein R., Shahmansouri N., Mogk J.P.M., Herrera S., Gault D., Brudy F., Lee M., Calin L., Lay Herrera T., Szkurlat D. (2025). Experiential space analysis: Scoring tranquil, social, and explorative places in habitable buildings. Front. Sustain. Cities.

[359] French D.J., Holroyd K.A., Pinell C., Malinoski P.T., O’donnell F., Hill K.R. (2000). Perceived self-efficacy and headache-related disability. Headache J. Head Face Pain.

[360] Peck K.R., Smitherman T.A. (2015). Mediator variables in headache research: Methodological critique and exemplar using self-efficacy as a mediator of the relationship between headache severity and disability. Headache J. Head Face Pain.

[361] Matsuzawa Y., Lee Y.S.C., Fraser F., Langenbahn D., Shallcross A., Powers S., Lipton R., Simon N., Minen M. (2019). Barriers to behavioral treatment adherence for headache: An examination of attitudes, beliefs, and psychiatric factors. Headache: J. Head Face Pain.

[362] Biber D.D., Ellis R. (2017). The effect of self-compassion on the self-regulation of health behaviors: A systematic review. J. Health Psychol..

[363] Phillips W.J., Hine D.W. (2021). Self-compassion, physical health, and health behaviour: A meta-analysis. Health Psychol. Rev..

[364] Wang J., Drossaert C.H.C., Knevel M., Chen L., Bohlmeijer E.T., Schroevers M.J. (2025). The Mechanisms Underlying the Relationship Between Self-Compassion and Psychological Outcomes in Adult Populations: A Systematic Review. Stress Health.

[365] Hennessy E.A., Johnson B.T., Acabchuk R.L., McCloskey K., Stewart-James J. (2020). Self-regulation mechanisms in health behavior change: A systematic meta-review of meta-analyses, 2006–2017. Health Psychol. Rev..

[366] Steinmetz J.D., Seeher K.M., Schiess N., Nichols E., Cao B., Servili C., Cavallera V., Cousin E., Hagins H., Moberg M.E. (2024). Global, regional, and national burden of disorders affecting the nervous system, 1990–2021: A systematic analysis for the Global Burden of Disease Study 2021. Lancet Neurol..

[367] Lipton R.B., Manack Adams A., Buse D.C., Fanning K.M., Reed M.L. (2016). A Comparison of the Chronic Migraine Epidemiology and Outcomes (CaMEO) Study and American Migraine Prevalence and Prevention (AMPP) Study: Demographics and Headache-Related Disability. Headache.

[368] Vetvik K.G., MacGregor E.A. (2017). Sex differences in the epidemiology, clinical features, and pathophysiology of migraine. Lancet Neurol..

[369] Lateef T.M., Merikangas K.R., He J., Kalaydjian A., Khoromi S., Knight E., Nelson K.B. (2009). Headache in a national sample of American children: Prevalence and comorbidity. J. Child Neurol..

[370] Lipton R.B., Manack A., Ricci J.A., Chee E., Turkel C.C., Winner P. (2011). Prevalence and burden of chronic migraine in adolescents: Results of the chronic daily headache in adolescents study (C-dAS). Headache.

[371] Lu Y., Li Q.-Y., Gan L., You Y., Wang C.-D., Guo Z.-W., Shi J., Liu X.-Y. (2025). The global and regional burden and trends of migraine from 1990 to 2021: Global Burden of Disease Study 2021. Front. Neurol..

[372] Steiner T.J., Stovner L.J., Jensen R., Uluduz D., Katsarava Z., on behalf of Lifting the Burden: The Global Campaign against Headache (2020). Migraine remains second among the world’s causes of disability, and first among young women: Findings from GBD2019. J. Headache Pain.

[373] Brennan K.C., Pietrobon D. (2018). A Systems Neuroscience Approach to Migraine. Neuron.

[374] Chase B.A., Frigerio R., Rubin S., Franada T., Semenov I., Meyers S., Bergman-Bock S., Mark A., Freedom T., Marcus R. (2024). An Integrative Migraine Polygenic Risk Score Is Associated with Age at Onset But Not Chronification. J. Clin. Med..

[375] Kogelman L.J.A., Esserlind A.L., Francke Christensen A., Awasthi S., Ripke S., Ingason A., Davidsson O.B., Erikstrup C., Hjalgrim H., Ullum H. (2019). Migraine polygenic risk score associates with efficacy of migraine-specific drugs. Neurol. Genet..

[376] Seng E.K., Martin P.R., Houle T.T. (2022). Lifestyle factors and migraine. Lancet Neurol..

[377] Marmura M.J. (2018). Triggers, Protectors, and Predictors in Episodic Migraine. Curr. Pain Headache Rep..

[378] Ashina S., Terwindt G.M., Steiner T.J., Lee M.J., Porreca F., Tassorelli C., Schwedt T.J., Jensen R.H., Diener H.-C., Lipton R.B. (2023). Medication overuse headache. Nat. Rev. Dis. Primers.

[379] Rosignoli C., Ornello R., Onofri A., Caponnetto V., Grazzi L., Raggi A., Leonardi M., Sacco S. (2022). Applying a biopsychosocial model to migraine: Rationale and clinical implications. J. Headache Pain.

[380] American Headache Society (2019). The American Headache Society Position Statement On Integrating New Migraine Treatments Into Clinical Practice. Headache J. Head Face Pain.

[381] Mungoven T.J., Henderson L.A., Meylakh N. (2021). Chronic Migraine Pathophysiology and Treatment: A Review of Current Perspectives. Front. Pain Res..

[382] Goadsby P.J., Holland P.R., Martins-Oliveira M., Hoffmann J., Schankin C., Akerman S. (2017). Pathophysiology of Migraine: A Disorder of Sensory Processing. Physiol. Rev..

[383] Puledda F., Silva E.M., Suwanlaong K., Goadsby P.J. (2023). Migraine: From pathophysiology to treatment. J. Neurol..

[384] Suzuki K., Suzuki S., Shiina T., Kobayashi S., Hirata K. (2022). Central Sensitization in Migraine: A Narrative Review. J. Pain Res..

[385] Yum J., Chu M.K. (2025). Unraveling the connections between migraine and psychiatric comorbidities: A narrative review. Brain Dev..

[386] Caponnetto V., Deodato M., Robotti M., Koutsokera M., Pozzilli V., Galati C., Nocera G., De Matteis E., De Vanna G., Fellini E. (2021). Comorbidities of primary headache disorders: A literature review with meta-analysis. J. Headache Pain.

[387] Sudat S.E., Jacobson A.S., Avins A.L., Lipton R.B., Pressman A.R. (2022). A population-health approach to characterizing migraine by comorbidity: Results from the Mindfulness and Migraine Cohort Study. Cephalalgia.

[388] Ailani J., Burch R.C., Robbins M.S., Board of Directors of the American Headache Society (2021). The American Headache Society Consensus Statement: Update on integrating new migraine treatments into clinical practice. Headache J. Head Face Pain.

[389] Eigenbrodt A.K., Ashina H., Khan S., Diener H.-C., Mitsikostas D.D., Sinclair A.J., Pozo-Rosich P., Martelletti P., Ducros A., Lantéri-Minet M. (2021). Diagnosis and management of migraine in ten steps. Nat. Rev. Neurol..

[390] Pellesi L., Garcia-Azorin D., Rubio-Beltrán E., Ha W.-S., Messina R., Ornello R., Petrusic I., Raffaelli B., Labastida-Ramirez A., Ruscheweyh R. (2024). Combining treatments for migraine prophylaxis: The state-of-the-art. J. Headache Pain.

[391] Martinelli D., De Icco R., Al-Khazali H.M., Ashina S., Diener H.-C., Dodd-Glover F., Goicochea M.T., Jenkins B., MaassenVanDenBrink A., Lee M.J. (2026). Advances in migraine prevention. Lancet Neurol..

[392] Charles A.C., Digre K.B., Goadsby P.J., Robbins M.S., Hershey A., Society T.A.H. (2024). Calcitonin gene-related peptide-targeting therapies are a first-line option for the prevention of migraine: An American Headache Society position statement update. Headache J. Head Face Pain.

[393] Hepp Z., Dodick D.W., Varon S.F., Gillard P., Hansen R.N., Devine E.B. (2015). Adherence to oral migraine-preventive medications among patients with chronic migraine. Cephalalgia.

[394] Hepp Z., Bloudek L.M., Varon S.F. (2014). Systematic review of migraine prophylaxis adherence and persistence. J. Manag. Care Pharm..

[395] Blumovich A., Gerson T., Connelly M., Wingert T., Jones G. (2025). The Real-World Evaluation of Remote Electrical Neuromodulation in Pediatric Migraines: A Preliminary Study. Children.

[396] Tana C., Garcia-Azorin D., Raffaelli B., Fitzek M.P., Waliszewska-Prosół M., Quintas S., Martelletti P. (2025). Neuromodulation in Chronic Migraine: Evidence and Recommendations from the GRADE Framework. Adv. Ther..

[397] Yuan H., Orr S.L., Al-Karagholi M.A.M., Ashina M., Cohen F., Diener H.-C., Dodick D.W., Jensen R.H., Marmura M.J., Martinelli D. (2025). International Headache society evidence-based guidelines on the use of non-invasive neuromodulation devices for the acute and preventive treatment of migraine. Cephalalgia.

[398] Chen X., Luo Y. (2023). Digital Therapeutics in Migraine Management: A Novel Treatment Option in the COVID-19 Era. J. Pain Res..

[399] Snipes C., Kuka A., Petrova M., Peters-Strickland T., Taraboanta L., Besedina O., Tepper S.J., Lakhan S. (2025). Efficacy and Safety of a First-in-Class Investigational Prescription Digital Therapeutic for Episodic Migraine (CT-132): Phase 3 Double-Blind, Randomized, Controlled Trial (P4-12.006). Neurology.

[400] Tana C., Raffaelli B., Moffa L., Götz J., Angerhöfer C. (2026). Digital and virtual interventions for migraine: A systematic review of randomized controlled trials. Cephalalgia.

[401] Lakhan S.E. (2025). Digital Step Therapy: A Smart Framework for Payer Adoption of Prescription Digital Therapeutics. Cureus.

[402] Noser A.E., Klages K.L., Gamwell K.L., Brammer C.N., Hommel K.A., Ramsey R.R. (2021). A systematic evaluation of primary headache management apps leveraging behavior change techniques. Cephalalgia.

[403] Blumenfeld A.M., Lipton R.B., Silberstein S., Tepper S.J., Charleston L.T., Landy S., Kuruvilla D.E., Manack Adams A. (2023). Multimodal Migraine Management and the Pursuit of Migraine Freedom: A Narrative Review. Neurol. Ther..

[404] Lipton R.B., Ailani J., Blumenfeld A.M. (2024). What Is Combination Treatment in Migraine? Moving Toward a Uniform Definition of a Familiar Principle. Neurol. Ther..

[405] Sverdlov O., van Dam J., Hannesdottir K., Thornton-Wells T. (2018). Digital Therapeutics: An Integral Component of Digital Innovation in Drug Development. Clin. Pharmacol. Ther..

[406] Biskupiak Z., Ha V.V., Rohaj A., Bulaj G. (2024). Digital Therapeutics for Improving Effectiveness of Pharmaceutical Drugs and Biological Products: Preclinical and Clinical Studies Supporting Development of Drug + Digital Combination Therapies for Chronic Diseases. J. Clin. Med..

[407] Onan D., Ekizoğlu E., Arıkan H., Taşdelen B., Özge A., Martelletti P. (2023). The Efficacy of Physical Therapy and Rehabilitation Approaches in Chronic Migraine: A Systematic Review and Meta-Analysis. J. Integr. Neurosci..

[408] Vandenbussche N., Moreno-Ajona D., Filippi M., Messina R. (2026). Chapter 7—Nonpharmacological approaches in migraine prevention: Neuromodulation, nutraceuticals, and behavioral approaches. Insights into Migraine Treatments.

[409] Blair H.A. (2023). Rimegepant: A Review in the Acute Treatment and Preventive Treatment of Migraine. CNS Drugs.

[410] Gao B., Sun N., Yang Y., Sun Y., Chen M., Chen Z., Wang Z. (2020). Safety and Efficacy of Fremanezumab for the Prevention of Migraine: A Meta-Analysis From Randomized Controlled Trials. Front. Neurol..

[411] Herd C.P., Tomlinson C.L., Rick C., Scotton W.J., Edwards J., Ives N.J., Clarke C.E., Sinclair A. (2019). Cochrane systematic review and meta-analysis of botulinum toxin for the prevention of migraine. BMJ Open.

[412] Raffaelli B., García-Azorín D., Boucherie D.M., Amin F.M., Deligianni C.I., Gil-Gouveia R., Kirsh S., Lampl C., Sacco S., Uluduz D. (2023). European Headache Federation (EHF) critical reappraisal and meta-analysis of oral drugs in migraine prevention—Part 3: Topiramate. J. Headache Pain.

[413] Jackson J.L., Kuriyama A., Kuwatsuka Y., Nickoloff S., Storch D., Jackson W., Zhang Z.-J., Hayashino Y. (2019). Beta-blockers for the prevention of headache in adults, a systematic review and meta-analysis. PLoS ONE.

[414] Tepper S.J., Rabany L., Cowan R.P., Smith T.R., Grosberg B.M., Torphy B.D., Harris D., Vizel M., Ironi A., Stark-Inbar A. (2023). Remote electrical neuromodulation for migraine prevention: A double-blind, randomized, placebo-controlled clinical trial. Headache J. Head Face Pain.

[415] Karimi N., Razian A., Heidari M. (2021). The efficacy of magnesium oxide and sodium valproate in prevention of migraine headache: A randomized, controlled, double-blind, crossover study. Acta Neurol. Belg..

[416] Domitrz I., Cegielska J. (2022). Magnesium as an Important Factor in the Pathogenesis and Treatment of Migraine-From Theory to Practice. Nutrients.

[417] Patel V., Akimbekov N.S., Grant W.B., Dean C., Fang X., Razzaque M.S. (2024). Neuroprotective effects of magnesium: Implications for neuroinflammation and cognitive decline. Front. Endocrinol..

[418] Maier J.A.M., Locatelli L., Fedele G., Cazzaniga A., Mazur A. (2023). Magnesium and the Brain: A Focus on Neuroinflammation and Neurodegeneration. Int. J. Mol. Sci..

[419] Song T.J., Chu M.K. (2021). Exercise in Treatment of Migraine Including Chronic Migraine. Curr. Pain Headache Rep..

[420] Kumar A., Bhatia R., Sharma G., Dhanlika D., Vishnubhatla S., Singh R.K., Dash D., Tripathi M., Srivastava M.V.P. (2020). Effect of yoga as add-on therapy in migraine (CONTAIN). Neurology.

[421] Wu Q., Liu P., Liao C., Tan L. (2022). Effectiveness of yoga therapy for migraine: A meta-analysis of randomized controlled studies. J. Clin. Neurosci..

[422] Nayar D., Mahapatro M., Nayar P. (2022). Role of Yoga as an Adjunct in the Management of Migraine Headache-Current Status and Future Indications. Int. J. Yoga.

[423] Sujan M.U., Inbaraj G., Raghavendra R.M., Vadiraja H.S., Madhu J.V., Mulakur S., Kisan R., Adoor M., Raghuram M.S., Nandakumar B. (2025). Yoga-based breathing and relaxation as adjunctive therapy for chronic migraine: A randomized controlled trial on clinical outcomes and autonomic regulation. Complement. Ther. Med..

[424] Sullivan D.P., Martin P.R., Boschen M.J. (2019). Psychological Sleep Interventions for Migraine and Tension-Type Headache: A Systematic Review and Meta-Analysis. Sci. Rep..

[425] Calhoun A.H., Ford S. (2007). Behavioral sleep modification may revert transformed migraine to episodic migraine. Headache.

[426] Minen M.T., Kaplan K., Akter S., Espinosa-Polanco M., Guiracocha J., Khanns D., Corner S., Roberts T. (2021). Neuroscience Education as Therapy for Migraine and Overlapping Pain Conditions: A Scoping Review. Pain Med..

[427] Pach D., Lysk S., Heinz P., Held U., Huber E., Scholler S., Dahlem M.A., Lysk M., Barth J., Icke K. (2025). A Prescribed Digital Health App and Number of Migraine Days: A Randomized Clinical Trial. JAMA Netw. Open.

[428] Tepper S.J., Cirillo J., Kim E., L’Italien G., Tweedie J.M., Lodaya K., Riley D., Pathan F., Antaki N., Nathanson B.H. (2023). The temporal trend of placebo response in migraine prevention from 1990 to 2021: A systematic literature review and meta-analysis with regression. J. Headache Pain.

[429] Ailani J., Lipton R.B., Goadsby P.J., Guo H., Miceli R., Severt L., Finnegan M., Trugman J.M. (2021). Atogepant for the Preventive Treatment of Migraine. N. Engl. J. Med..

[430] Lipton R.B., Goadsby P.J., Smith J., Schaeffler B.A., Biondi D.M., Hirman J., Pederson S., Allan B., Cady R. (2020). Efficacy and safety of eptinezumab in patients with chronic migraine: PROMISE-2. Neurology.

[431] Dodick D.W., Ashina M., Brandes J.L., Kudrow D., Lanteri-Minet M., Osipova V., Palmer K., Picard H., Mikol D.D., Lenz R.A. (2018). ARISE: A Phase 3 randomized trial of erenumab for episodic migraine. Cephalalgia.

[432] Bruijn N.R.A., Huessler E.M., MaassenVanDenBrink A., Fronczek R., Diener H.C., Ferrari M.D., Al-Hassany L. (2026). Placebo Response in Acute and Prophylactic Treatment of Migraine: A Systematic Review and Meta-Analysis Covering 36 Years of Research. Neurol. Clin..

[433] Sacco S., Ashina M., Diener H.-C., Haghdoost F., Lee M.J., Monteith T.S., Jenkins B., Peres M.F.P., Pozo-Rosich P., Ornello R. (2025). Setting higher standards for migraine prevention: A position statement of the International Headache Society. Cephalalgia.

[434] Rosen N., Pearlman E., Ruff D., Day K., Jim Nagy A. (2018). 100% Response Rate to Galcanezumab in Patients with Episodic Migraine: A Post Hoc Analysis of the Results From Phase 3, Randomized, Double-Blind, Placebo-Controlled EVOLVE-1 and EVOLVE-2 Studies. Headache J. Head Face Pain.

[435] Lipton R.B., Cohen J.M., Bibeau K., Galic M., Seminerio M.J., Ramirez Campos V., Halker Singh R.B., Ailani J. (2020). Reversion From Chronic Migraine to Episodic Migraine in Patients Treated With Fremanezumab: Post Hoc Analysis From HALO CM Study. Headache.

[436] Moskatel L.S., Slusky D.J.G. (2025). The state of insurance coverage of calcitonin gene-related peptide-targeted medications and its impact on the implementation of the American Headache Society’s 2024 consensus statement: An interrupted time-series analysis. Headache.

[437] Varnado O.J., Brady B., Zagar A., Robles Y., Céilleachair A.Ó., Hoyt M. (2025). Health care resource utilization and direct costs incurred over 12 months by patients with migraine initiating self-injectable calcitonin gene-related peptide monoclonal antibodies: A US real-world study. J. Manag. Care Spec. Pharm..

[438] Pozo-Rosich P., Caronna E., Sacco S., Peres M.F.P., Ashina S., Özge A., Ahmed F., Velez-Jimenez M.K., Jenkins B., Wang S.-J. (2025). Early treatment in migraine—A call to shift prevention from attacks to disease progression: A position statement from the International Headache Society. Cephalalgia.

[439] Yabroff K.R., Zhao J., Halpern M.T., Fedewa S.A., Han X., Nogueira L.M., Zheng Z., Jemal A. (2021). Health Insurance Disruptions and Care Access and Affordability in the U.S. Am. J. Prev. Med..

[440] Jung M.J., Kanegi S.L., Rosen N.L. (2024). Treating the Uninsured and Underinsured with Migraine in the USA. Curr. Pain Headache Rep..

[441] Majersik J.J., Ahmed A., Chen I.H.A., Shill H., Hanes G.P., Pelak V.S., Hopp J.L., Omuro A., Kluger B., Leslie-Mazwi T. (2021). A Shortage of Neurologists—We Must Act Now. Neurology.

[442] Tekin B.H. (2026). A Comparative Evaluation of Multimodal Generative AI as an Early-Stage Biophilic Design Assistant. Buildings.

[443] Chen J., Shao Z., Zheng X., Zhang K., Yin J. (2024). Integrating aesthetics and efficiency: AI-driven diffusion models for visually pleasing interior design generation. Sci. Rep..

[444] Rui L., Firzan M. (2025). Emotional Design of Interior Spaces: Exploring Challenges and Opportunities. Buildings.

[445] Albayrak-Kutlay Y., Bengisu M., Ergül E. (2026). Biofeedback-Informed Assessment of Biophilic Interior Variables: A 23 IVR Factorial Study in Design Studio Interiors. Architecture.

[446] Loughnane C., Laiti J., O’Donovan R., Dunne P.J. (2025). Systematic review exploring human, AI, and hybrid health coaching in digital health interventions: Trends, engagement, and lifestyle outcomes. Front. Digit. Health.

[447] Serra H., Zavattaro C., Eid M., Farina P., Abbatescianna D., Cirillo E., Gammeri R., Celi L., Scariot V., Ricci R. (2025). Biophilic interventions in real and virtual environments reduce stress during cognitively demanding tasks. Sci. Rep..

[448] Aristizabal S., Byun K., Porter P., Clements N., Campanella C., Li L., Mullan A., Ly S., Senerat A., Nenadic I.Z. (2021). Biophilic office design: Exploring the impact of a multisensory approach on human well-being. J. Environ. Psychol..

[449] Ríos-Rodríguez M.L., Testa Moreno M., Moreno-Jiménez P. (2023). Nature in the Office: A Systematic Review of Nature Elements and Their Effects on Worker Stress Response. Healthcare.

